# Effectiveness of Telematic Behavioral Techniques to Manage Anxiety, Stress and Depressive Symptoms in Patients with Chronic Musculoskeletal Pain: A Systematic Review and Meta-Analysis

**DOI:** 10.3390/ijerph19063231

**Published:** 2022-03-09

**Authors:** Ferran Cuenca-Martínez, Luis Suso-Martí, Aida Herranz-Gómez, Clovis Varangot-Reille, Joaquín Calatayud, Mario Romero-Palau, María Blanco-Díaz, Cristina Salar-Andreu, Jose Casaña

**Affiliations:** 1 Exercise Intervention for Health Research Group (EXINH-RG), Department of Physiotherapy, University of Valencia, 46010 Valencia, Spain; ferran.cuenca@uv.es (F.C.-M.); aidahergo10@gmail.com (A.H.-G.); clovis.varangotreille@gmail.com (C.V.-R.); joaquin.calatayud@uv.es (J.C.); jose.casana@uv.es (J.C.); 2 Department of Developmental and Educational Psychology, University of Valencia, 46010 Valencia, Spain; mromeropalau1998@gmail.com; 3 Surgery and Medical Surgical Specialties Department, Faculty of Medicine and Health Sciences, University of Oviedo, 33003 Oviedo, Spain; blancomaria@uniovi.es; 4 Universidad CEU Cardenal Herrera, CEU Universities, 46115 Valencia, Spain; cristina.salar@uchceu.es

**Keywords:** telerehabilitation, behavior, depression, anxiety, stress

## Abstract

Anxiety, depressive symptoms and stress have a significant influence on chronic musculoskeletal pain. Behavioral modification techniques have proven to be effective to manage these variables; however, the COVID-19 pandemic has highlighted the need for an alternative to face-to-face treatment. We conducted a search of PubMed, the Cumulative Index to Nursing and Allied Health Literature (CINAHL), Web of Science, APA PsychInfo, and Psychological and Behavioural Collections. The aim was to assess the effectiveness of telematic behavioral modification techniques (e-BMT) on psychological variables in patients with chronic musculoskeletal pain through a systematic review with meta-analysis. We used a conventional pairwise meta-analysis and a random-effects model. We calculated the standardized mean difference (SMD) with the corresponding 95% confidence interval (CI). Forty-one randomized controlled trials were included, with a total of 5018 participants. We found a statistically significant small effect size in favor of e-BMT in depressive symptoms (n = 3531; SMD = −0.35; 95% CI −0.46, −0.24) and anxiety (n = 2578; SMD = −0.32; 95% CI −0.42, −0.21) with low to moderate strength of evidence. However, there was no statistically significant effect on stress symptoms with moderate strength of evidence. In conclusion, e-BMT is an effective option for the management of anxiety and depressive symptoms in patients with chronic musculoskeletal pain. However, it does not seem effective to improve stress symptoms.

## 1. Introduction

The COVID-19 pandemic has shaken our lives and jeopardized the treatment of countless patients with chronic pain [1,2]. Chronic pain patients have shown a significant increase in their perceived pain in comparison with the pre-pandemic period [3], as well as an increase in depressive symptoms, anxiety, loneliness, tiredness and catastrophizing [3]. Nearly half of a sample of 2423 chronic pain patients had moderate to severe psychological distress [4]. The worsening of mental health in patients with chronic pain is not without consequences; these variables have been linked to higher pain catastrophizing, pain-related fear and avoidance, and a higher risk of misuse of opioids [5,6].

These patients need follow-up, a close relationship with health professionals and appropriate treatment, but social distancing prevents them from doing so [1]. Chronic pain patients had higher self-isolation than participants without pain during the pandemic [3]. Because it does not require being physically present, telerehabilitation, or the therapeutic use of technological devices, has been recommended for chronic pain management worldwide [2]. Over the last few decades, behavioral modification techniques (BMT) have showed to be effective in the management of psychological variables in chronic pain patients [7,8]. However, it is not clear if telematic BMT (e-BMT) is also effective to improve psychological variables and if it is as effective as in-person BMT. Some previous systematic reviews have assessed the effect of telerehabilitation based on BMT on variables such as pain intensity, disability, disease impact, physical function, pain-related fear of movement, and psychological distress [9,10,11,12], showing promising results.

The primary aim of this systematic review with meta-analysis was to evaluate the effectiveness of e-BMT compared with usual care/waiting list or in-person BMT in psychological variables. Secondly, we aimed to sub-analyze the results by intervention parameters and diagnostic conditions. The main reason for the secondary aim was because the “BMT” label includes a large range of interventions and so we can isolate effects by intervention or by clinical entities.

## 2. Materials and Methods

This systematic review and meta-analysis was performed according to the Preferred Reporting Items for Systematic Reviews and Meta-Analysis (PRISMA) 2020 statement [13]. This systematic review was registered prospectively in an international database (PROSPERO), where it can be accessed (CRD42021278086).

### 2.1. Search Strategy

The search strategy of this systematic review is the same as another systematic review from our research group on this topic, which is currently under review. The search for studies was performed using Medline (PubMed), the Cumulative Index to Nursing and Allied Health Literature (CINAHL), Web of Science, APA PsychInfo, and Psychological and Behavioural Collections, from inception to (30) August 2021. In addition, we manually checked the references of the studies included in the review and checked the studies included in systematic reviews related to this topic. The search was also adapted and performed in Google Scholar due to its capacity to search for relevant articles and grey literature [14]. No restrictions were applied to any specific language. The different search strategies used are detailed in Appendix A.1.

Two independent reviewers (CVR and FCM) conducted the search using the same methodology, and the differences were resolved by consensus moderated by a third reviewer (JCG). We used Rayyan software to organize studies, assess studies for eligibility and remove duplicates [15].

### 2.2. Study Eligibility Criteria

The selection criteria used in this systematic review and meta-analysis were based a Population, Intervention, Control, Outcomes, and Study design framework (PICOS). We included randomized controlled trials that have applied e-BMT through a technology device (Website, online, telephone or mobile application). The intervention could be applied alone or embedded with another treatment, only if the control group contains only the additional treatment. Control group could be usual care, waiting list, no intervention, or in-person equivalent BMT. The participants selected for the studies were patients older than 18 years with any kind of chronic musculoskeletal disorder. The participants’ gender was irrelevant. We excluded patients with musculoskeletal pain due to oncologic or traumatic process. The measures used to assess the results were depressive symptoms, anxiety, and stress. Time of measurement was restrained to post-treatment results.

### 2.3. Selection Process and Data Extraction

The two phases of studies selection (title/abstract screening and full-text evaluation) were realized by two independent reviewers (CVR and FCM). First, they assessed the relevance of the studies regarding the study questions and aims, based on information from the title, abstract, and keywords of each study. If there was no consensus or the abstracts did not contain sufficient information, the full text was reviewed. In the second phase of the analysis, the full text was used to assess whether the studies met all the inclusion criteria. Differences between the two independent reviewers were resolved by a consensus process moderated by a third reviewer (JCG). Data described in the results were extracted by means of a structured protocol that ensured that the most relevant information was obtained from each study [16].

### 2.4. Risk of Bias and Methodological Quality Assessment

The Risk Of Bias 2 (RoB 2) tool was used to assess randomized trials [17]. It covers a total of 5 domains: (1) Bias arising from the randomization process, (2) Bias due to deviations from the intended interventions, (3) Bias due to missing outcome data, (4) Bias in measurement of the outcome, (5) Bias in selection of the reported result. The study will be categorized as having (a) low risk of bias if all domains shown low risk of bias, (b) some concerns if one domain is rated with some concerns without any with high risk of bias, and (c) high risk of bias, if one domain is rated as having high risk of bias or multiple with some concerns.

The studies’ methodological quality was assessed using the PEDro scale [18], which assesses the internal and external validity of a study and consists of 11 criteria. The methodological criteria were scored as follows: yes (1 point), no (0 points), or do not know (0 points). The PEDro score for each selected study provided an indicator of the methodological quality (9–10 = excellent; 6–8 = good; 4–5 = fair; 3–0 = poor) [19]. We used the data obtained from the PEDro scale to map the results of the quantitative analyses.

Two independent reviewers (LSM and FCM) examined the quality and the risk of bias of all the selected studies using the same methodology. Disagreements between the reviewers were resolved by consensus with a third reviewer (JCG). Concordance between the results (inter-rater reliability) was measured using Cohen’s kappa coefficient (κ) as follows: (1) κ > 0.7 indicated a high level of agreement between assessors; (2) κ = 0.5–0.7 indicated a moderate level of agreement; and (3) κ < 0.5 indicated a low level of agreement [20].

### 2.5. Quality of Evidence

The quality of evidence analysis was based on classifying the results into levels of evidence according to the Grading of Recommendations, Assessment, Development and Evaluation (GRADE) framework, which is based on 5 domains: study design, imprecision, indirectness, inconsistency, and publication bias [21]. The assessment of the 5 domains was conducted according to GRADE criteria [22,23]. Evidence was categorized into the following 4 levels accordingly: (a) *High quality.* Further research is very unlikely to change our confidence in the effect estimate. All 5 domains are also met. (b) *Moderate quality.* Further research is likely to have an important impact on our confidence in the effect estimate and might change the effect estimate. One of the 5 domains is not met. (c) *Low quality.* Further research is very likely to have a significant impact on our confidence in the effect estimate and is likely to change the estimate. Two of the 5 domains are not met. (d) *Very low quality.* Any effect estimates highly uncertain. Three of the 5 domains are not met [22,23].

For the risk of bias domain, the recommendations were downgraded one level in the event there was an uncertain or high risk of bias and serious limitations in the effect estimate (more that 25% of the participants were from studies with high risk of bias, as measured by the RoB 2 scale). In terms of inconsistency, the recommendations were downgraded one level when the point estimates varied widely among studies, the confidence intervals showed minimal overlap or when the I^2^ was substantial or large (greater than 50%). For the indirectness domain, recommendations were downgraded when severe differences in interventions, study populations or outcomes were found. (The recommendations were downgraded in the absence of direct comparisons between the interventions of interest or when there are no key outcomes, and the recommendation is based only on intermediate outcomes or if more than 50% of the participants were outside the target group.) For the imprecision domain, the recommendations were downgraded one level if there were fewer than 300 participants for the continuous data. Finally, the recommendations were downgraded due to strong influence of publication bias if the results changed significantly after adjusting for publication bias.

### 2.6. Data Synthesis

The statistical analysis was conducted using *RStudio* software version 1.4.1717, which is based on *R* software version 4.1.1 [24,25]. To compare the outcomes reported by the studies, we calculated the standardized mean difference (SMD), as Hedge’s g, over time and the corresponding 95% confidence interval (CI) for the continuous variables. It was interpreted as described by Hopkins et al. [26]. If necessary, CI and standard error (SE) were converted into standard deviation (SD) [27]. The estimated SMDs were interpreted as described by Hopkins et al. [26]; that is, we considered an SMD of 4.0 to represent an extremely large clinical effect, 2.0–4.0 represented a very large effect, 1.2–2.0 represented a large effect, 0.6–1.2 represented a moderate effect, 0.2–0.6 represented a small effect, and 0.0–0.2 represented a trivial effect.

We used the same inclusion criteria for the systematic review and the meta-analysis and included 3 additional criteria: (1) In the results, there was detailed information regarding the comparative statistical data of the exposure factors, therapeutic interventions, and treatment responses; (2) the intervention was compared with a similar control group; and (3) data on the analyzed variables were represented in at least 3 studies.

As we pooled different treatments, we could not assume that there was a unique true effect. So, we anticipated between-study heterogeneity and used a random-effects model to pool effect sizes. In order the calculate the heterogeneity variance τ^2^, we used the Restricted Maximum Likelihood Estimator as recommended for continuous outcomes [28,29]. To calculate the confidence interval around the pooled effect, we used Knapp–Hartung adjustments [30,31].

We estimated the degree of heterogeneity among the studies using Cochran’s Q statistic test (a *p*-value < 0.05 was considered significant), the inconsistency index (I^2^) and the prediction interval (PI) based on the between-study variance τ^2^ [26]. Cochran’s Q test allows us to assess the presence of between-study heterogeneity [32]. Despite its common use to assess heterogeneity, the I^2^ index only represents the percentage of variability in the effect sizes not caused by a sampling error [33]. Therefore, as recommended, we additionally report PIs. The PIs are an equivalent to standard deviation and represent a range within which the effects of future studies are expected to fall based on current data [33,34].

To detect the presence of outliers that could potentially influence the estimated pooled effect and assess the robustness of our results, we applied an influence analysis based on the leave-one-out method [35]. If the study’s results had an important influence on the pooled effect, we conducted a sensitivity analysis removing it or them. We additionally ran a drapery plot, which is based on *p*-value functions and gives us the *p*-value curve for the pooled estimate for all possible alpha levels [36].

To detect publication bias, we performed a visual evaluation of the Doi plot and the funnel plot [37], seeking asymmetry. We also performed a quantitative measure of the Luis Furuya Kanamori (LFK) index, which has been shown to be more sensitive than the Egger test in detecting publication bias in a meta-analysis of a low number of studies [38]. An LFK index within ±1 represents no asymmetry, exceeding ±1 but within ±2 represents minor asymmetry, and exceeding ±2 involves major asymmetry. If there was significant asymmetry, we applied a small-study effect method to correct for publication bias using the Duval and Tweedie trim and fill method [39].

For the qualitative analysis, we reported the between-group mean difference (MD) with the 95% CI for the outcomes of interest. If it was not reported by the authors, we calculated it [40].

## 3. Results

### 3.1. Descriptions of the Studies

From the 749 studies initially detected, a total of 41 RCTs were included [41,42,43,44,45,46,47,48,49,50,51,52,53,54,55,56,57,58,59,60,61,62,63,64,65,66,67,68,69,70,71,72,73,74,75,76,77,78,79,80,81]. The PRISMA 2020 flow chart is detailed in Appendix A.2. We included 5018 participants with a mean age ranging from 33.7 to 65.8 years. The patients were mostly women (N = 3631, 72.4%) diagnosed with chronic back pain [47,52,72,79,80], chronic low back pain [41,55], unspecific chronic pain [43,51,53,56,59,67,68,69,70,71,73,74,75,76,81], fibromyalgia [42,46,48,49,58,63,66], headache [44,60,61,78], rheumatic disorders [45,57,62,64], or others [50,54,65]. Details of the participant’s characteristics and studies are shown in Appendix A.3.

The studies compared online cognitive-behavioral therapy [42,43,45,46,47,54,55,59,63,70,72,79,80,81], acceptance and commitment therapy [56,58,70,71,73,76], self-management [52,59,62,66,67,68,69,77], mindfulness therapy [61,65,70,72,76], or other e-BMT [41,44,48,49,50,53,57,60,64,74,75,78], against most frequently waiting list [43,44,46,48,51,54,56,57,60,62,64,68,71,72,74,75,77,79,80,81], usual care [42,45,47,49,52,55,58,59,61,63,66,67,69,70,73,78], or in-person intervention [50,63,76]. The intervention duration ranged between a single day [65] and 6 months [41,50,62,66,78]. The details of the interventions are described in Appendix A.4 using the Behavior Change Technique Taxonomy (v1) [82].

### 3.2. Methodological Quality and Risk of Bias

**Figure 1:**
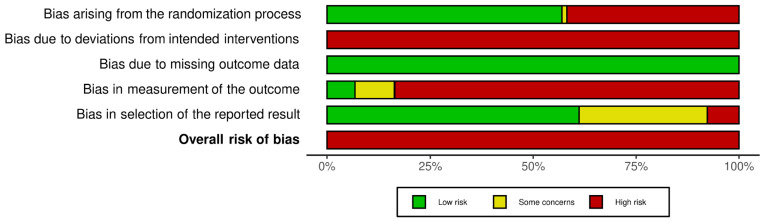
Risk of bias graph according to the Risk of Bias 2 tool.

According to the PEDro scale, 30 were evaluated as having good [41,42,43,44,45,46,47,48,49,50,51,55,56,58,59,62,63,64,65,66,68,70,71,72,73,75,76,77,78,80] and 11 as having fair methodological quality [52,53,54,57,60,61,67,69,74,79,81] (Appendix A.5). The inter-rater reliability of the methodological quality assessment between assessors was high (κ = 0.823). According to the Rob 2 scale, all the studies have a high risk of bias (100%) (Figure 1 and Appendix A.6). The inter-rater reliability of the risk of bias assessment between assessors was high (κ = 0.884).

### 3.3. Qualitative Synthesis

Four studies compared e-BMT with in-person BMT. They applied CBT [47,63], ACT [76] or person-centered intervention [50]. Two found non-statistically significant differences between groups for depressive symptoms (n = 253; MD = 0.24, 95% CI −2.32 to 2.80 [47] and MD = −0.51, 95% CI −2.42 to 1.40 [76]); however, Vallejo et al. found statistically significant between-group differences post-treatment in favor of e-BMT (n = 40; MD = −5.06, 95% CI −7.39 to −2.73) [63]. One found a non-statistically significant difference between groups for anxiety (n = 128; MD = −4.20, 95% CI −10.58 to 2.17) [76] and one found a non-statistically significant difference between groups for stress (n = 109; MD = −2.76, 95% CI −5.94 to 1.28) [50].

### 3.4. Quantitative Synthesis

#### 3.4.1. Depressive Symptoms

**Figure 2:**
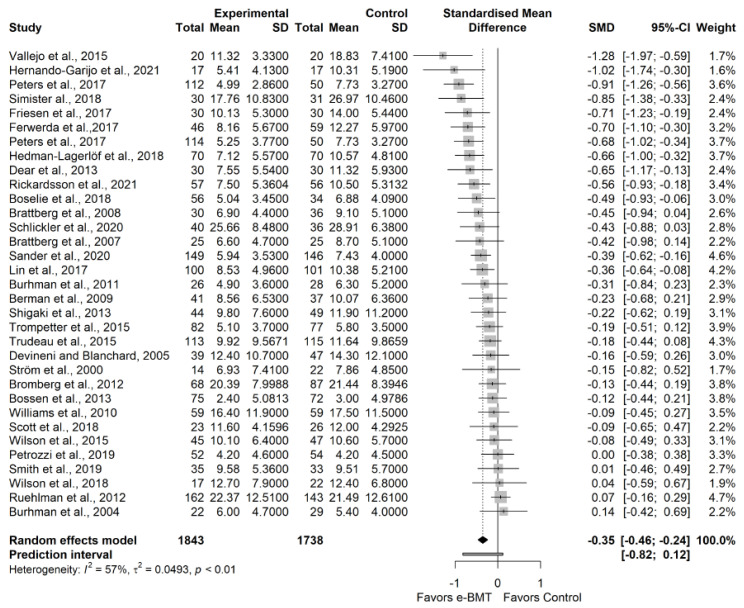
Sensitivity analysis of the depressive symptoms variable for telematic behavioral modification techniques against usual care or waiting list. Negative results favor the intervention group. The small boxes with the squares represent the point estimate of the effect size and sample size. The lines on either side of the box represent a 95% confidence interval (CI). e-BMT: Telematic Behavioral Modification Techniques.

According to the influence analyses, we conducted a sensitivity analysis without Dear et al. [43]. We found a statistically significant small effect size (32 RCTs; n = 3531; SMD = −0.35; 95% CI −0.46, −0.24) of e-BMT on depressive symptoms compared with usual care or waiting list, with significant heterogeneity (Q = 74.06 (*p* < 0.01); I^2^ = 57% (36%, 71%); PI −0.82, 0.12) and a low strength of evidence (Figure 2). Since PI crosses zero, we cannot be confident that future studies will not find contradictory results; however, the results appear to be robust to different *p*-value functions. With respect to the presence of publication bias, the funnel and Doi plots show an asymmetrical pattern, demonstrating minor asymmetry (LFK index = −1.62). When the sensitivity analysis is adjusted for publication bias, there is still a small significant effect. Statistical analyses are detailed in Appendix A.7. Subgroup analyses are detailed in Table 1a.

#### 3.4.2. Anxiety

**Figure 3:**
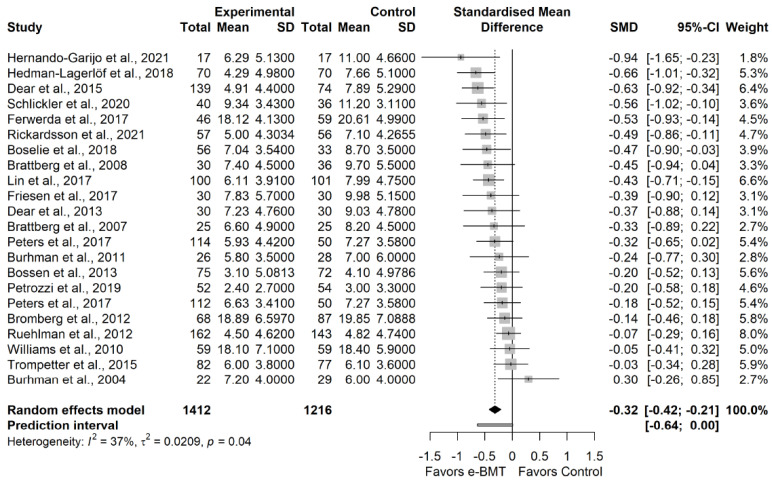
Sensitivity analysis of the anxiety variable for telematic behavioral modification techniques against usual care or waiting list. Negative results favor the intervention group. The small boxes with the squares represent the point estimate of the effect size and sample size. The lines on either side of the box represent a 95% confidence interval (CI). e-BMT: Telematic Behavioral Modification Techniques.

According to the influence analyses, we conducted a sensitivity analysis without Trudeau et al. [62]. We found a statistically significant small effect size (21 RCTs; n = 2578; SMD = −0.32; 95% CI −0.42, −0.21) of e-BMT on anxiety compared with usual care or waiting list, with significant heterogeneity (Q = 33.47 (*p* = 0.04); I^2^ = 37% (0%, 63%); PI −0.64, 0.00) and a moderate strength of evidence (Figure 3). Since PI crosses zero, we cannot be confident that future studies will not find contradictory results; however, the results appear to be robust to different *p*-value functions. With respect to the presence of publication bias, the funnel and Doi plots show a symmetrical pattern, demonstrating no asymmetry (LFK index = −0.48). Statistical analyses are detailed in Appendix A.8. Subgroup analyses are detailed in Table 1b.

#### 3.4.3. Stress

**Figure 4:**
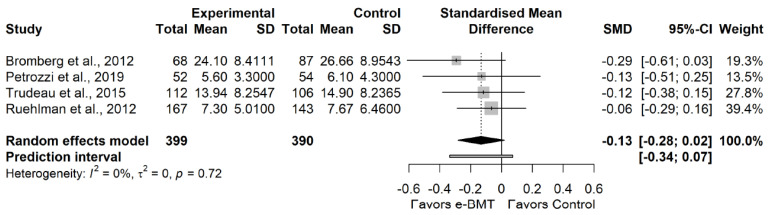
Statistical analysis of the stress variable for telematic behavioral modification techniques against usual care or waiting list. Negative results favor intervention group. The small boxes with the squares represent the point estimate of the effect size and sample size. The lines on either side of the box represent a 95% confidence interval (CI). e-BMT: Telematic Behavioral Modification Techniques.

We found no statistically significant effect size (4 RCTs; n = 789; SMD = −0.13; 95% CI −0.28, 0.02) of e-BMT on stress compared with usual care or waiting list, with significant heterogeneity (Q = 1.33 (*p* = 0.72); I^2^ = 0% (0%, 85%); PI −0.34, 0.07) and a moderate strength of evidence (Figure 4). Since PI crosses zero, we cannot be confident that future studies will not find contradictory results. With respect to the presence of publication bias, the funnel and Doi plots show an asymmetrical pattern, demonstrating minor asymmetry (LFK index = −1.55). When the sensitivity analysis is adjusted for publication bias, there is no influence on the estimated effect. Statistical analyses are detailed in Appendix A.9.

GRADE’s overall strength of the evidence is detailed in Table 2.

## 4. Discussion

The primary aim of this systematic review with meta-analysis was to evaluate the effectiveness of e-BMT compared with usual care/waiting list or in-person BMT in terms of psychological variables. Secondly, we aimed to sub-analyze the results by intervention parameters and diagnostic conditions. The main results found that e-BMT seems to be an effective option for the management of anxiety and depressive symptoms in patients with musculoskeletal conditions causing chronic pain but not to improve stress symptoms. e-BMT does not seem to provide greater improvement than in-person BMT for psychological variables.

Several research studies have been published and have shown similar results to those found in this review with meta-analysis with regard to depressive and anxiety symptoms. For example, the rapid review conducted by Varker et al. [83] aimed to evaluate the effectiveness of e-BMT (by videoconference) and also through conventional mobile phone calls for people with high levels of anxiety and depression. The main results showed that both rehabilitation modalities produced significant positive results in terms of decreasing the levels of both psychological variables. In addition to this, the review conducted by McCall et al. [84] found that delivering psychological telematic interventions resulted in a significant decrease in depressive symptoms but could not be proven to be effective in comparison to face-to-face psychological intervention. Anxiety symptoms could not be assessed. This work included few studies, so the results have to be interpreted with caution.

In addition to being a possible alternative to in-person treatment, e-BMT appears to be a cost-effective technique compared to in-person BMT. De Boer et al. compared e-BMT and in-person BMT in patients with chronic pain and found that the costs of online CBT were EUR 199 lower than in-person BMT [85]. Similarly, Aspvall et al. found that after 6 months of follow-up in children and adolescents with obsessive compulsive disorder, there was a difference of USD 1688 in favor of e-BMT [86]. Healthcare systems and guidelines should seriously consider implementing e-BMT in the management of patients with musculoskeletal disorders causing chronic pain.

### 4.1. Practical Implication

Concerning clinical implications, the results showed good results in favor of e-BMT. This gives us an effective treatment window in the COVID-19 era, so we are going to have a greater impact on patients with persistent pain. In addition, there is a decentralization of interventions, which may have some positive effects such as improving and increasing adherence to treatments due to easier accessibility, as well as lowering barriers to access or facilitating follow-up. Future studies should also focus on longer follow-ups to see this effectiveness and evaluate variables such as motivation or adherence to chronic pain treatments. Finally, telemedicine rehabilitation may lead to lower costs for both patients and therapists, which may reduce waiting lists for clinical treatments.

### 4.2. Limitations

We found limited evidence for depressive symptoms; true effects might be different from our estimated effects. We found the presence of publication bias for depressive and stress symptoms; however, adjustments did not influence the results. All the studies have a high risk of bias; results should be interpreted cautiously. Future studies should improve their design quality to enhance our trust in their results. We have pooled together different BMT and conditions. However, we also provided sub-analyses where depressive symptoms and anxiety are analyzed by treatment and by condition.

## 5. Conclusions

e-BMT is an effective option for the management of anxiety and depressive symptoms in patients with musculoskeletal conditions causing chronic pain and should be introduced when in-person intervention is not possible. However, it does not seem effective to improve stress symptoms.

## Figures and Tables

**Table 1:** Subgroup analysis.

Outcomes Sub = Analysis	N Studies	SMD	Lower Limit 95%CI	Upper Limit 95% CI	Q	I^2^
**(a) Depressive Symptoms**—Treatment						
ACT	5	−0.39	−0.71	−0.07	6.38	37%
CBT	11	−0.46	−0.73	−0.19	29.21	66%
Positive Psychology	2	−0.61	−1.77	0.55	0.45	0%
Self-management	8	−0.12	−0.26	0.03	6.30	0%
Other types of treatment	7	−0.30	−0.58	−0.03	11.19	46%
**Depressive Symptoms**—Chronic Musculoskeletal disorder						
Back pain	5	−0.24	−0.53	0.05	5.58	28%
Fibromyalgia	7	−0.66	−1.01	−0.31	14.16	58%
Headache	3	−0.14	−0.19	−0.09	0.02	0%
Rheumatic disorders	4	−0.28	−0.68	0.12	5.85	49%
Unspecified chronic pain	13	−0.33	−0.51	−0.15	36.61	65%
**Depressive Symptoms**—Added to usual care treatment? (Y/N)						
Only e-BMT	24	−0.34	−0.46	−0.22	52.26	54%
e-BMT added to usual care	8	−0.41	−0.80	−0.03	21.79	68%
**Depressive Symptoms**—Intervention duration						
Between 1 and 6 weeks	6	−0.02	−0.17	0.12	2.44	0%
Between 7 and 11 weeks	18	−0.46	−0.61	−0.31	36.70	51%
12 weeks and more	8	−0.26	−0.50	−0.03	12.54	44%
**Depressive Symptoms**—Methodological Quality according to the PEDro scale						
Fair methodological quality	7	−0.18	−0.43	0.07	10.86	45%
Good methodological quality	25	−0.39	−0.52	−0.26	54.08	54%
**(b) Anxiety**—Treatment						
ACT	3	−0.31	−0.93	0.31	4.75	58%
CBT	10	−0.31	−0.50	−0.12	14.71	39%
Positive psychology	2	−0.37	-1.28	0.53	0.28	0%
Self-Management	3	−0.20	−0.70	0.30	2.34	15%
Other types of treatment	4	−0.41	−0.97	0.14	8.43	64%
**Anxiety**—Chronic Musculoskeletal disorder						
Unspecific back pain	3	−0.09	−0.75	0.58	2.43	18%
Fibromyalgia	5	−0.45	−0.85	−0.05	8.17	51%
Headache	1	−0.14	−0.85	0.18	N/A	N/A
Rheumatic disorders	2	−0.35	-2.47	1.77	1.67	40%
Unspecified chronic pain	10	−0.33	−0.47	−0.19	16.12	38%
**Anxiety**—Intervention duration						
1 to 6 weeks	2	0.02	-1.96	2.01	1.41	29%
7 to 11 weeks	13	−0.41	−0.50	−0.31	10.34	0%
12 weeks and more	6	−0.25	−0.56	0.06	9.13	45%
**Anxiety**—Added to usual care treatment? (Y/N)						
Only e-BMT	17	−0.34	−0.45	−0.22	26.85	37%
e-BMT added to usual care	4	−0.19	−0.59	0.22	4.95	39%
**Anxiety**—Methodological Quality according to the PEDro scale						
Fair methodological quality	5	−0.18	−0.40	0.04	6.61	24%
Good methodological quality	16	−0.37	−0.49	−0.24	22.28	33%

Abbreviatures: ACT: Acceptance and Commitment therapy; CBT: Cognitive-behavioral therapy; CI: Confidence interval; e-BMT: Telematic behavioral techniques; N/A: Not Applicable; SMD: Standardized mean difference; Y/N: Yes.

**Table 2:** GRADE’s overall strength of the evidence.

Certainty Assessment							No. of Participants		Effect	Certainty
Outcome (No. of Studies)	Study Design	Risk of Bias	Inconsistency	Indirectness	Imprecision	Publication Bias	e-BMT	Control	Absolute (95% CI)	
Depressive symptoms (n = 32)	RCT	Serious	Serious	Not serious	Not serious	Not serious	1843	1688	−0.35 (−0.46; −0.24)	**Low** **⊕⊕**
Anxiety (n = 21)	RCT	Serious	Not Serious	Not serious	Not serious	Not serious	1412	1166	−0.32 (−0.42; −0.21)	**Moderate** **⊕⊕⊕**
Stress (n = 4)	RCT	Serious	Not serious	Not serious	Not serious	Not serious	399	390	−0.13 (−0.28; 0.02)	**Moderate** **⊕⊕⊕**

CI: Confidence interval, e-BMT: Telematic Behavioral Modification Techniques, RCT: Randomized controlled trial.

**Figure A1:**
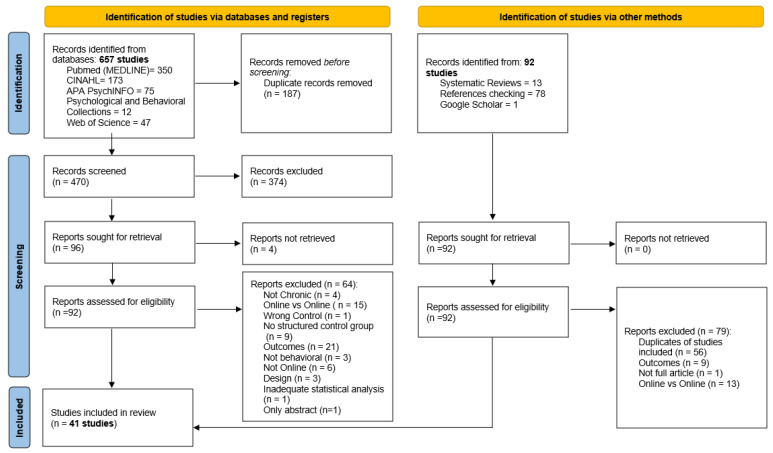
PRISMA Flowchart of studies selection.

**Figure A2:**
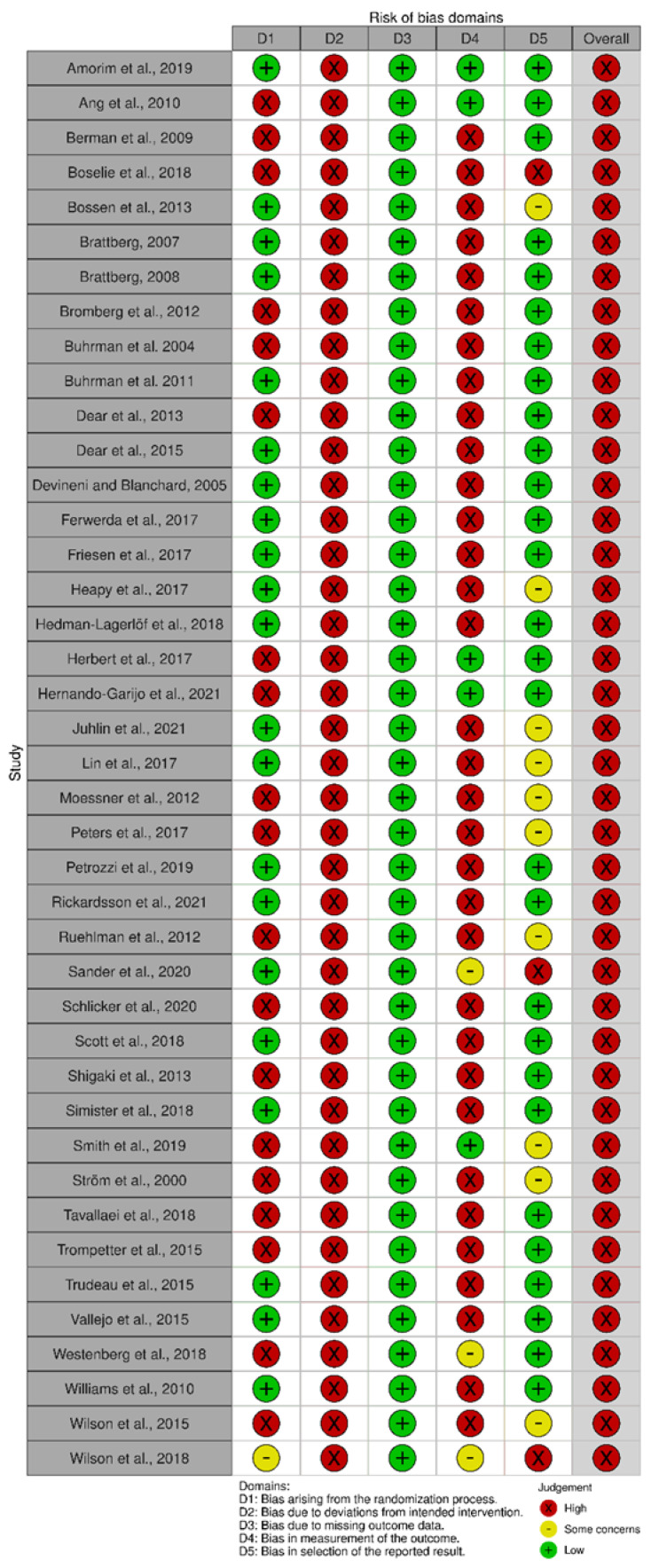
Risk of Bias 2.

**Figure A3:**
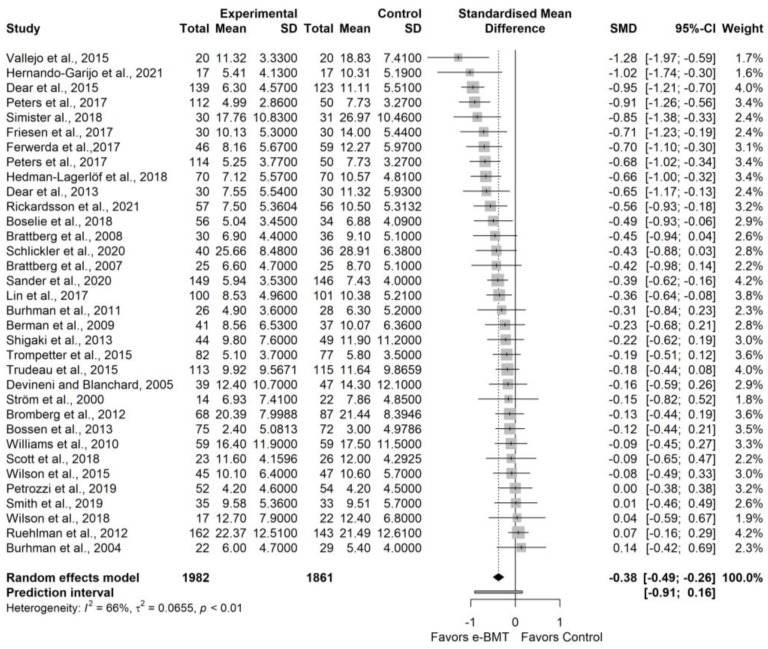
Forest plot of all the studies.

**Figure A4:**
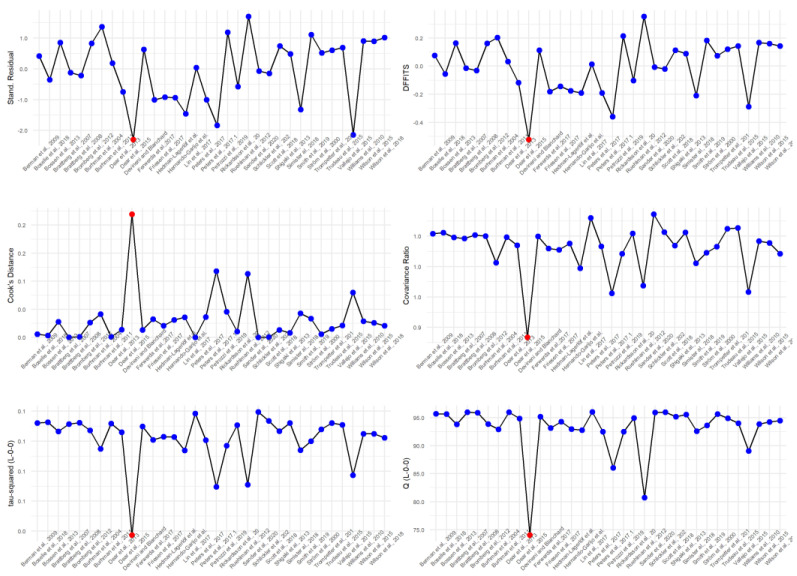
Influence analyses of all the studies.

**Figure A5:**
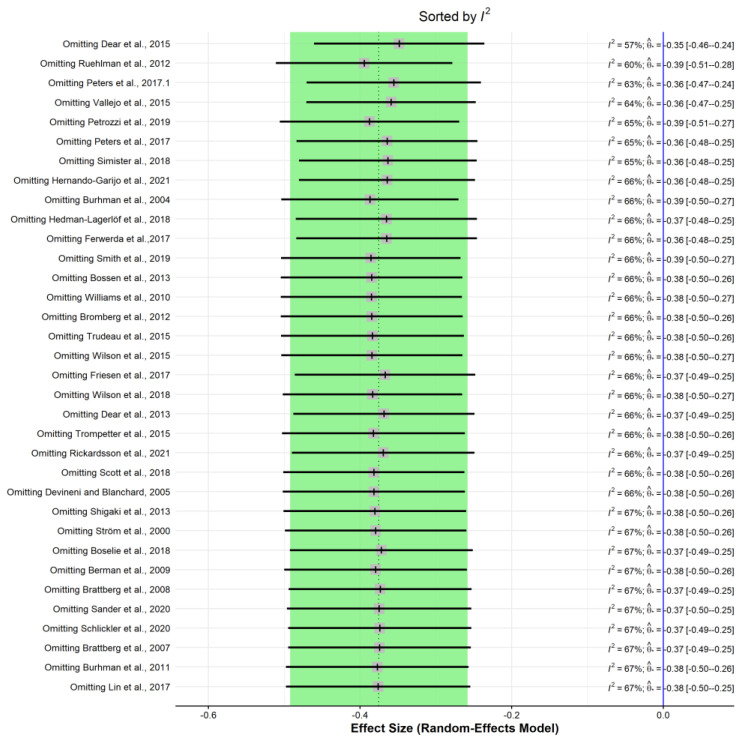
Leave-one-out figure of all the studies.

**Figure A6:**
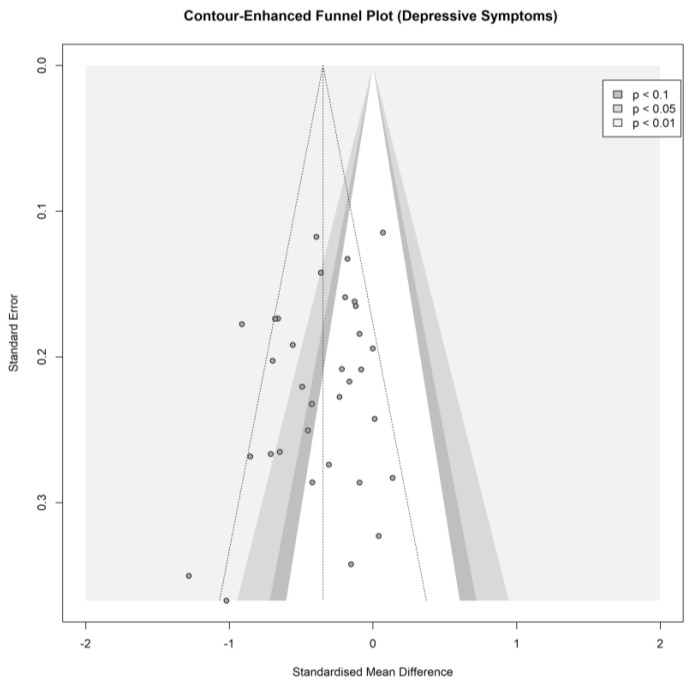
Contour-enhanced funnel plot of the studies included in the sensitivity analysis.

**Figure A7:**
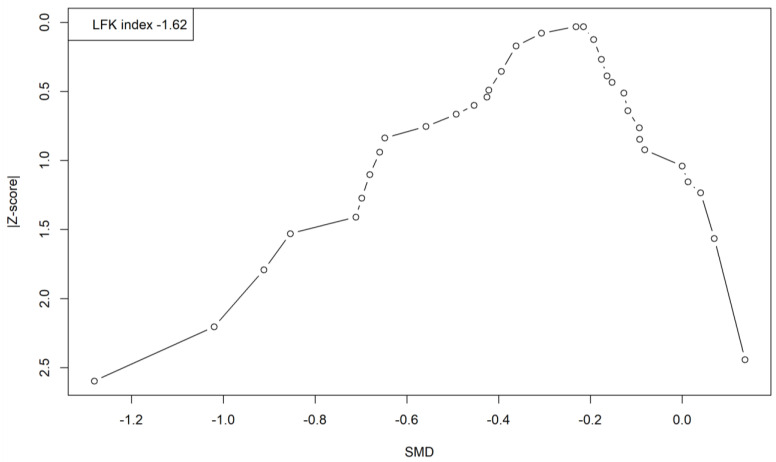
Doi plot and LFK index of the studies included in the sensitivity analysis.

**Figure A8:**
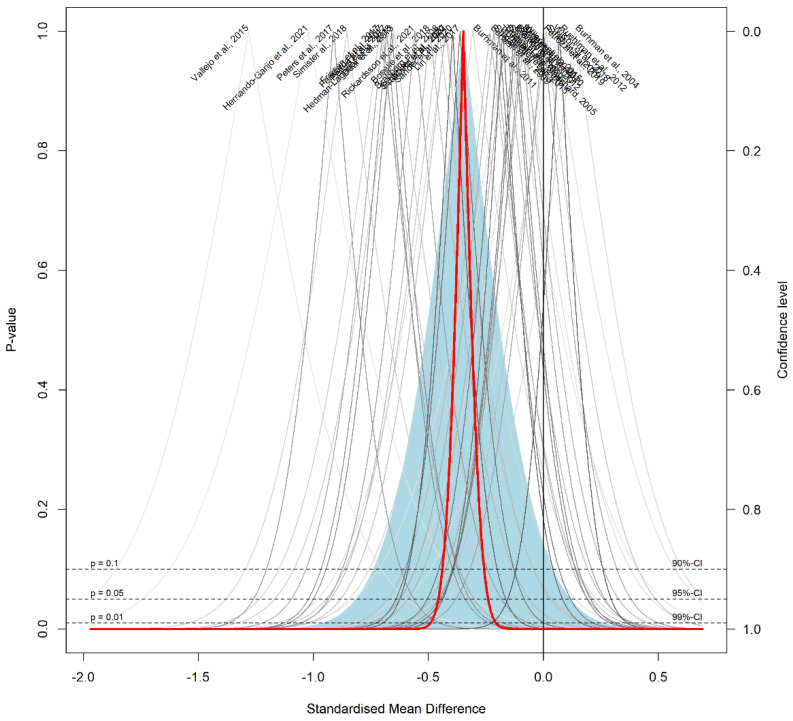
Drapery plot of the studies included in the sensitivity analysis.

**Figure A9:**
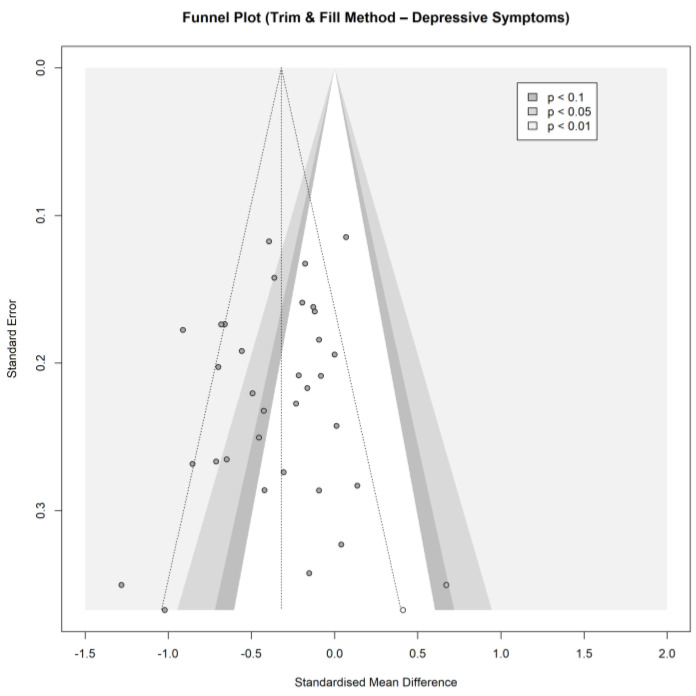
Contour-enhanced funnel plot of the studies included in the sensitivity analysis and the studies filled to adjust for publication bias.

**Figure A10:**
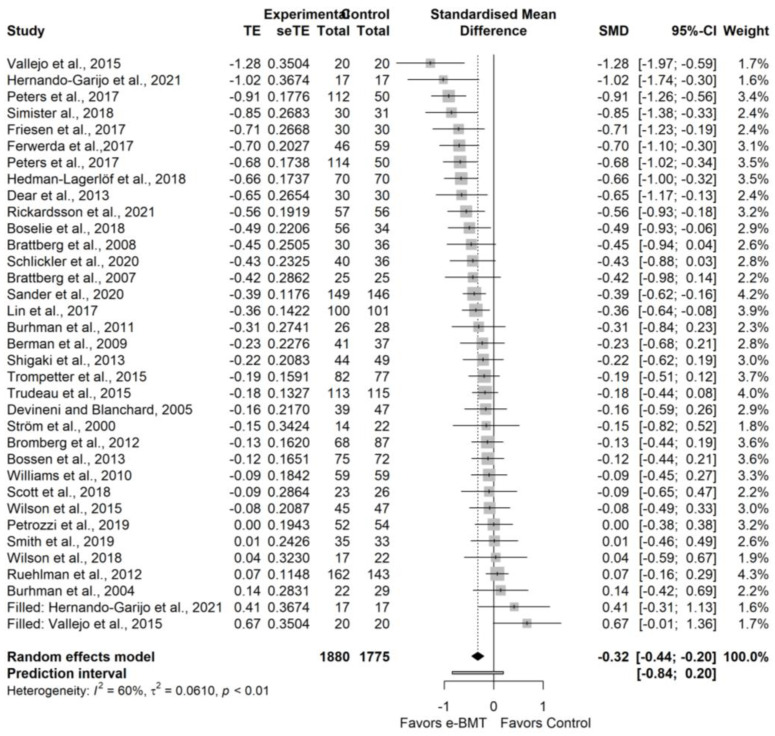
Forest plot of the studies included in the sensitivity analysis and the studies filled to adjust for publication bias.

**Figure A11:**
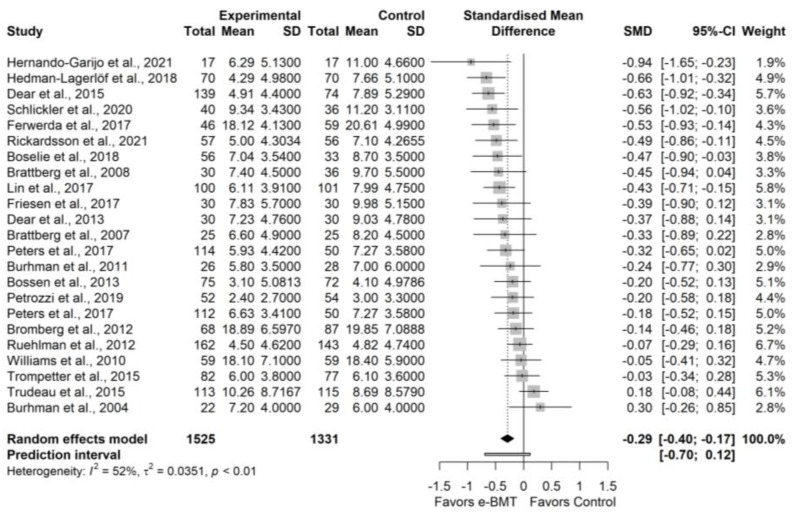
Forest plot with all the studies.

**Figure A12:**
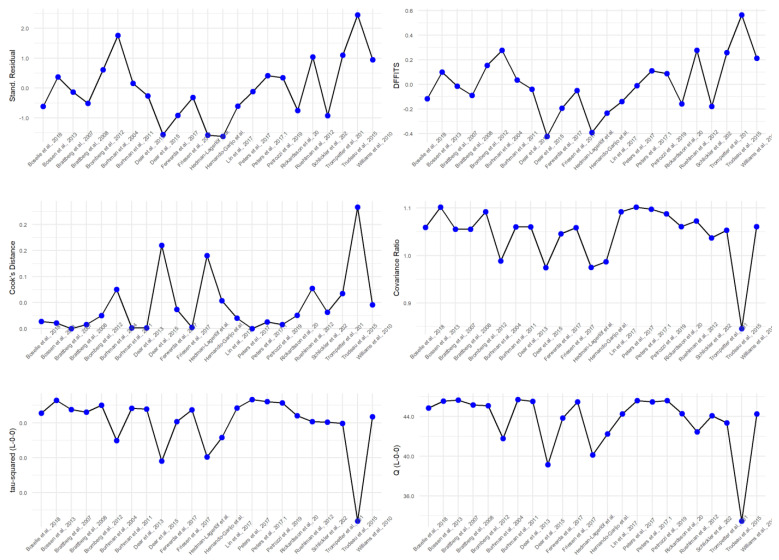
Influence analyses of all the studies.

**Figure A13:**
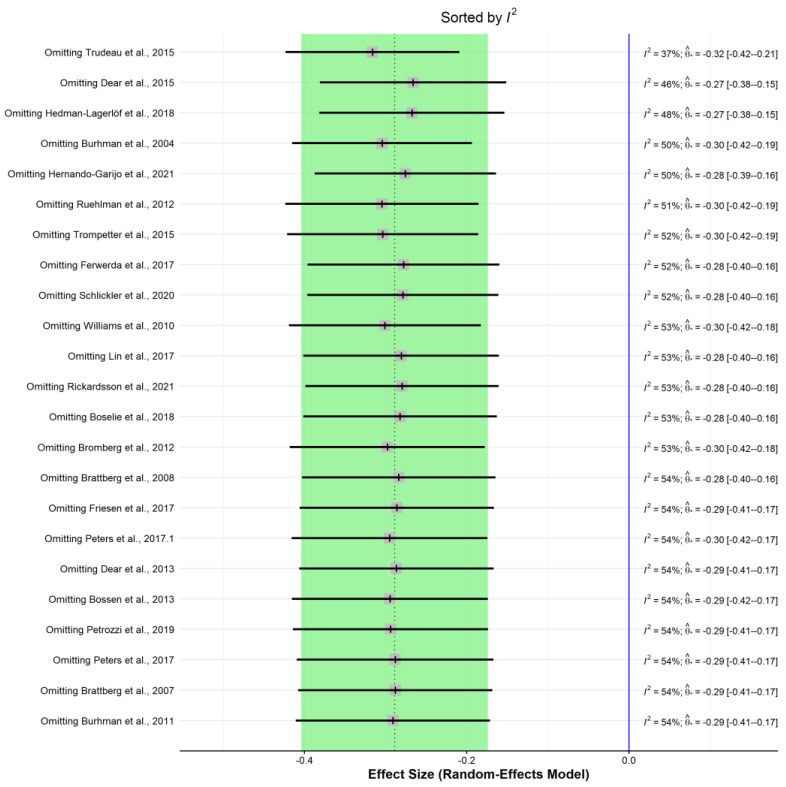
Leave-one-out figure of all the studies.

**Figure A14:**
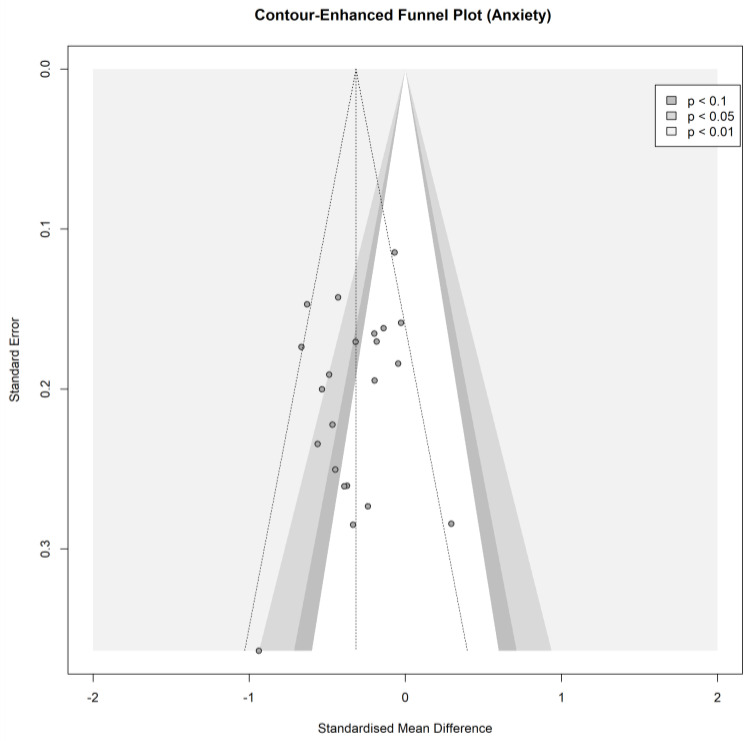
Contour-enhanced funnel plot of the studies included in the sensitivity analysis.

**Figure A15:**
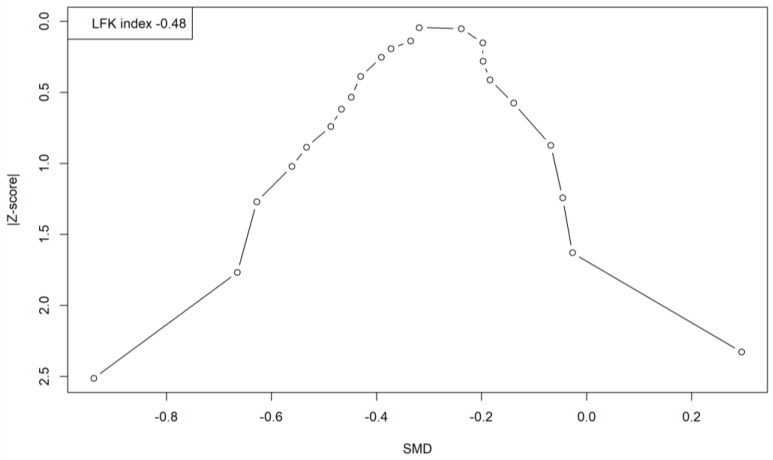
Doi plot and LFK index of the studies included in the sensitivity analysis.

**Figure A16:**
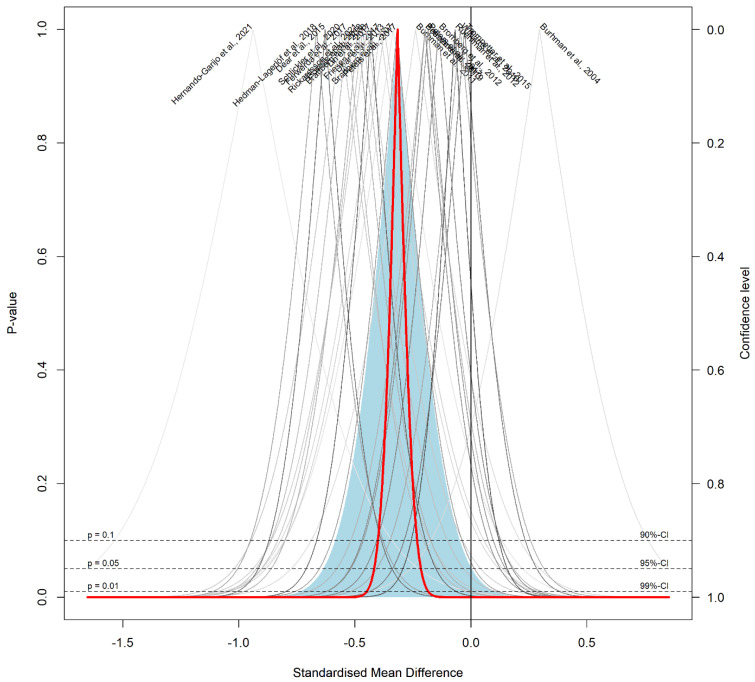
Drapery plot of the studies included in the sensitivity analysis.

**Figure A17:**
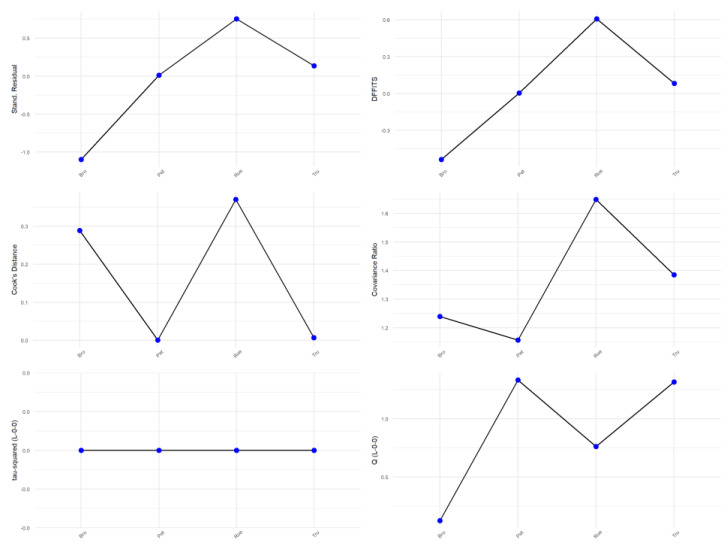
Influence analyses of all the studies.

**Figure A18:**
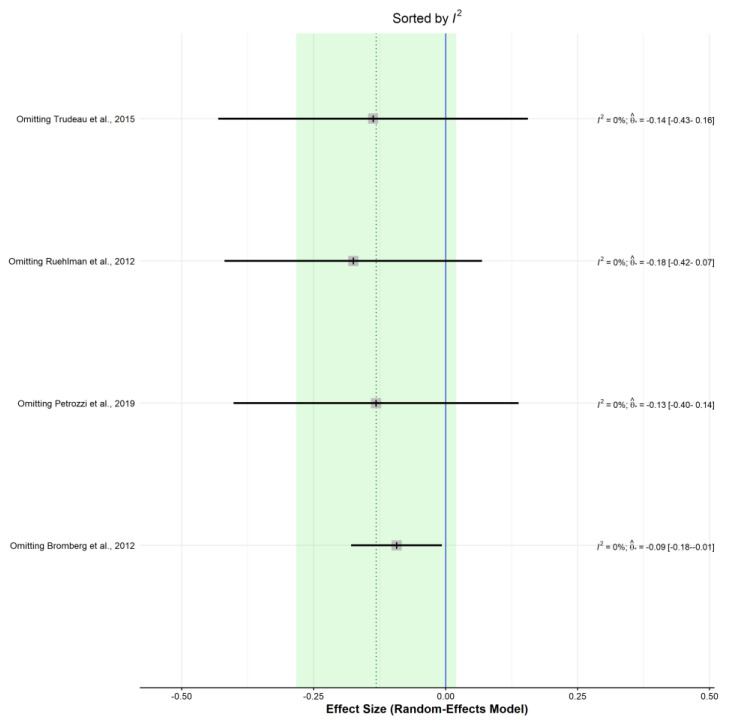
Leave-one-out figure of all the studies.

**Figure A19:**
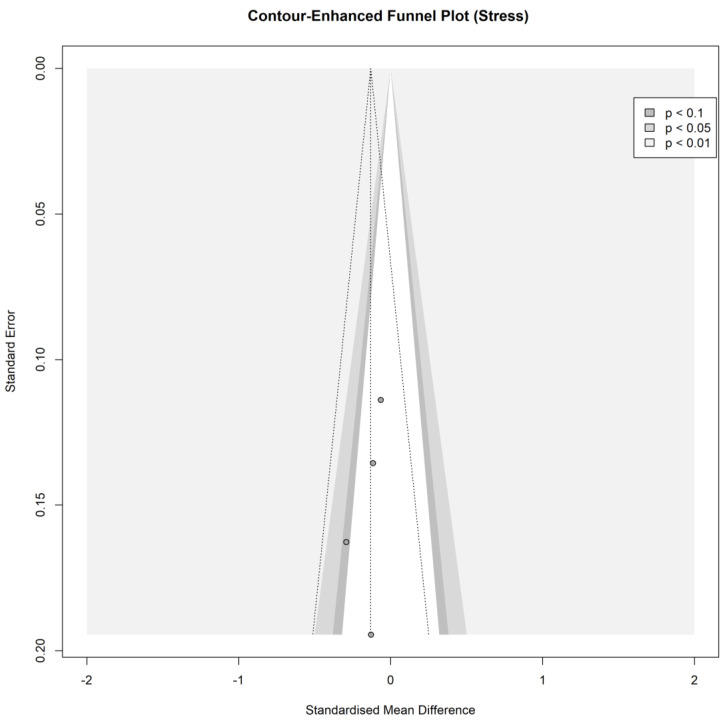
Contour-enhanced funnel plot of all the studies.

**Figure A20:**
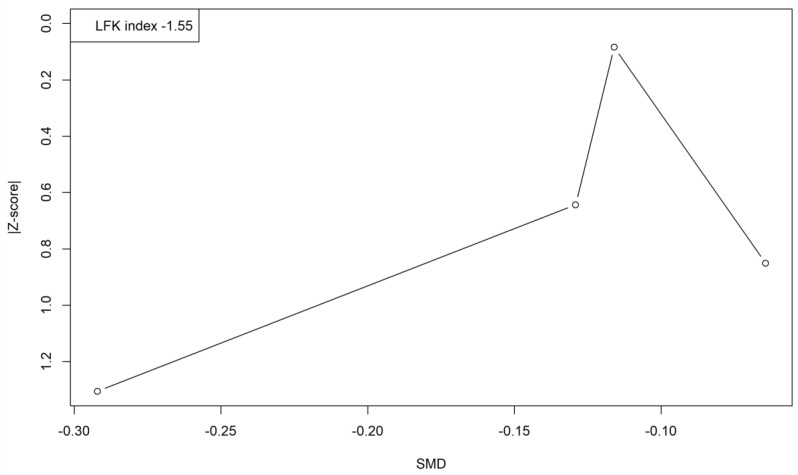
Doi plot and LFK index of all the studies.

**Figure A21:**
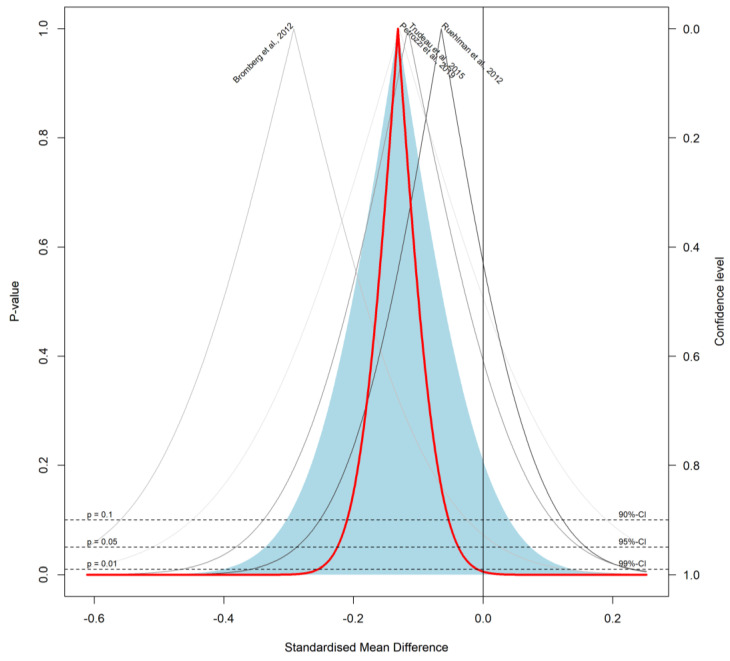
Drapery plot of all the studies.

**Figure A22:**
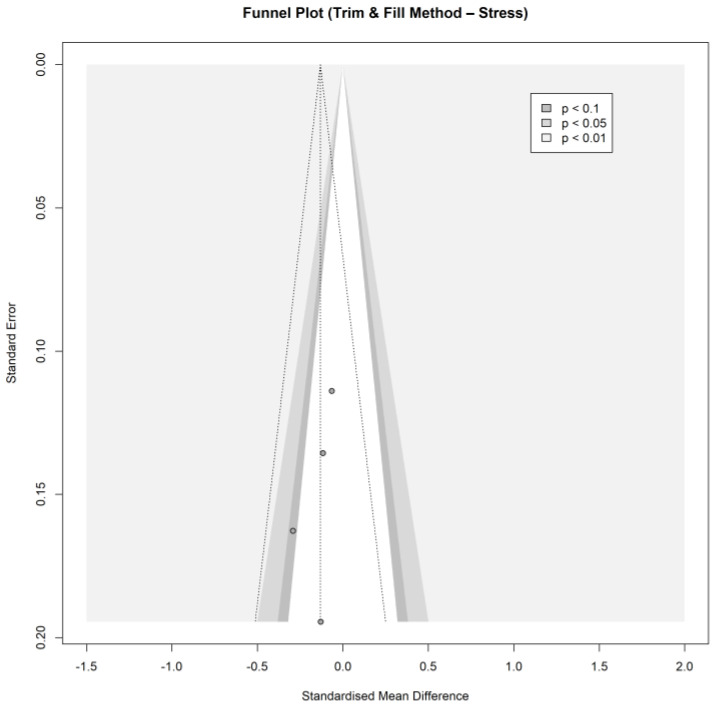
Contour-enhanced funnel plot of the studies included in the sensitivity analysis and the studies filled to adjust for publication bias. The trim and fill method did not add any study.

**Figure A23:**
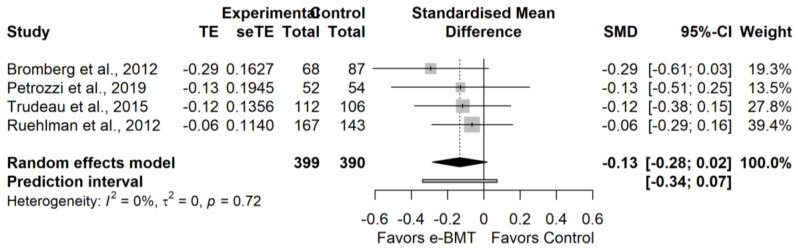
Forest plot of the studies included in the sensitivity analysis and the studies filled to adjust for publication bias The trim and fill method did not add any study.

## Appendix A

### Appendix A.1

Pubmed—350 results.

((“Web”) OR (“ehealth”) OR (“mhealth”) OR (“remote treatment”) OR (“digital treatment”) OR (“Mobile Applications”[MesH]) OR (“Software”[Mesh]) OR (“Online”) OR (“Telephone”) OR (“Cell phone”[MesH]) OR (“eTherapy”) OR (“Internet”) OR (“Online”) OR (“Telerehabilitation”) OR (“Internet-Based Intervention”[MesH]) OR (“Telerehabilitation”[MesH]) OR (Telemedicine[MesH])) AND ((“Chronic Pain”) OR (“Chronic Pain”[Mesh])) AND (randomized controlled trial[pt] OR controlled clinical trial[pt] OR randomized[tiab] OR placebo[tiab] OR clinical trials as topic[mesh:noexp] OR randomly[tiab] OR trial[ti] NOT (animals[mh] NOT humans [mh]) NOT (“protocol”) NOT (“Review”)).

CINAHL—173 results.

(web or internet or online or mobile or remote treatment or digital treatment or Internet-Based Intervention or Telerehabilitation or Telemedicine) AND (chronic pain or persistent pain or long term pain or long-term pain) AND (randomized controlled trials or rct or randomised control trials) NOT (systematic review or meta-analysis or literature review or review of literature) NOT (pediatric or child or children or infant or adolescent)

Psychology and Behavioral Sciences Collection (EBSCO)—12 results.

(web or internet or online or mobile or remote treatment or digital treatment or Internet-Based Intervention or Telerehabilitation or Telemedicine or) AND (chronic pain or persistent pain or long term pain or long-term pain) AND (randomized controlled trials or rct or randomised control trials) NOT (systematic review or meta-analysis or literature review or review of literature) NOT (pediatric).

APA PsychINFO—75 results.

(web or websites or internet or online or Online Therapy or mobile or Mobile Applications or remote treatment or digital treatment or Digital Interventions or Internet-Based Intervention or Telerehabilitation or Telemedicine) AND (chronic pain or persistent pain or long term pain or long-term pain) AND (randomized controlled trials or rct or randomised control trials) NOT (systematic review or meta-analysis or literature review or review of literature) NOT (pediatric or child or children or infant or adolescent).

Web of science—49 studies.

TI = (Web OR eearth OR melth OR remote treatment OR digital treatment OR Mobile Applications OR Software OR Online OR Telephone OR Cell phone OR estherapy OR Internet OR Online OR Telerehabilitation OR Internet-Based Intervention OR Telerehabilitation OR Telemedicine) AND TI = (Chronic pain) AND TI = (randomi?ed controlled trial* OR rct).

Google Scholar.

(“web” OR “online” OR “internet” OR “mobile” OR “telerehabilitation” OR “telemedicine”) AND [allintitle:”chronic pain” OR “persistent pain”] AND (“randomized controlled trial” OR “randomised controlled trial OR “RCT”)-review.

### Appendix A.3. Details of the Studies Included in the Systematic Review

**Table A1:** Details of the Studies Included in the Systematic Review.

Authors, Year Design Country	Participants Sample Size (n) Age (Mean (SD)) Gender *Condition*	Intervention Modality *Format*	Comparator	Outcomes	Results
**Amorim et al., 2019** **Pilot RCT** **Australia**	N = 68 58.3 (13.4) yrs 50%F/50%M *Chronic LBP*	Activity tracker and monitoring application. + Telephone follow-up *Mobile application*	Advice to stay active and booklet	*Depressive symptoms, anxiety and stress: DASS*	No significant differences on the outcomes.
**Ang et al., 2010** **RCT** **USA**	N = 32 48.9 (10.9) yrs 100%F *Fibromyalgia*	CBT + Usual care *Telephone*	Usual care	*Depressive symptoms: PHQ-8*	Non-significant difference on depressive symptoms (*p* = 0.8).
**Berman et al., 2009** **RCT** **USA**	N = 89 65.8 (N/R) yrs 87%F/13%M *Unspecified chronic pain*	Self-care intervention *Internet-based*	No intervention	*Depressive symptoms: CES-D 10*	Small non-significant effect on anxiety and depressive symptoms only in self-care group (*p* > 0.05).
**Boselie et al., 2018** **RCT** **The Netherlands**	N = 33 N/R yrs N/R%F/N/R%M *Unspecified chronic pain*	Positive psychology *Internet-based*	Waiting list	*Depressive symptoms and anxiety: HADS*	Significant main effect of PPI condition on anxiety (*p* = 0.02) and depressive symptoms (*p* = 0.01).
**Bossen et al., 2013** **RCT** **The Netherlands**	N = 199 62.0 (5.7) yrs 65%F/35%M *Knee and hip OA*	Behavior-graded activity program *Internet-based*	Waiting list	*Anxiety and depressive symptoms: HADS*	At the end of the intervention, intervention group showed less anxiety (*p* = 0.007). Other outcomes showed no significant differences.
**Brattberg, 2007** **RCT** **Sweden**	N = 60 47.0 (8.0) yrs 90%F/10%M *Unspecified chronic pain*	Support/self-help group about pain. Internet-based videos or CDs	Waiting list	*Anxiety and Depressive symptoms: HADS*	Intervention group showed a higher improvement in depressive symptoms over time (*p* = 0.04) but not in anxiety (*p* = 0.4).
**Brattberg, 2008** **RCT** **Sweden**	N = 66 43.8 (8.8) yrs 100%F *Fibromyalgia*	Emotional freedom techniques Internet-based	Waiting list	*Anxiety and Depressive symptoms: HADS*	Intervention group showed a statistically significant time*group interaction in depressive symptoms (*p* = 0.02) and anxiety (*p* = 0.03).
**Bromberg et al., 2012** **RCT** **USA**	N = 189 42.6 (11.5) yrs 89%F/11%M *Chronic migraine*	Structured behavior changes program +Usual care Internet-based	Usual care	*Depressive symptoms, anxiety and stress: DASS-21*	Intervention group showed a higher improvement in depressive symptoms (*p* = 0.008) and stress (*p* = 0.04), but not on anxiety.
**Buhrman et al., 2004** **RCT** **Sweden**	n = 56 44.6 (10.4) yrs 63%F/37%M *Chronic back pain*	Online CBT + Relaxation with CDs + Telephone calls about goals Internet-based	Waiting list	*Anxiety and depressive symptoms: HADS*	There was no significant main effects difference on anxiety and depressive symptoms.
**Buhrman et al., 2011** **RCT** **Sweden**	N = 54 43.2 (9.8) yrs 69%F/32%M *Chronic back pain*	Online CBT Internet-based	Waiting list	*Anxiety and depressive symptoms: HADS*	There were no significant differences between group for anxiety and depressive symptoms.
**Dear et al., 2013** **RCT** **Australia**	N = 63 49.0 (13) yrs 85%F/15%M *Unspecified chronic pain*	Online CBT Internet-based	Waiting list	*-Depressive symptoms: PHQ-9* *-Anxiety: GAD-7*	Intervention had a significantly higher post-treatment improvement in depressive symptoms (*p* < 0.001), anxiety (*p* < 0.001).
**Dear et al., 2015** **RCT** **Australia**	N = 490 50 (13) yrs 80%F/20%M *Unspecified chronic pain*	G1: Online CBT + Regular online contact G2: Online CBT + optimal online contact G3: Online CBT Internet-based	Waiting list	*-Depressive symptoms: PHQ-9* *-Anxiety: GAD-7*	Intervention groups had significantly lower scores than waiting list for depressive symptoms and anxiety (*p* < 0.001) post-treatment.
**Devineni and Blanchard, 2005** **RCT** **USA**	N = 86 42.2 (11.9) yrs 62%F/38%M *Chronic migraine and/or tension-type headache*	Behavioral headache-related intervention Internet-based	Waiting list	*Depressive symptoms: CES-D*	There was no statistically significant difference for depressive symptoms (*p* = 0.11) and anxiety (*p* = 0.20).
**Ferwerda et al.,2017** **RCT** **The Netherlands**	N = 133 56.4(10) yrs 64%F/36%M *Rheumatoid arthritis*	CBT Internet-based	Usual care	*-Depressive symptoms: BDI* *-Negative mood and Anxiety: IRGL*	Intervention group report a larger decrease in anxiety (*p* < 0.001) and depressed mood (*p* < 0.001) than control group.
**Friesen et al., 2017** **RCT** **Canada**	N = 60 48.0 (11.0) yrs 95%F/5%M *Fibromyalgia*	CBT + Telephone calls Internet-based	Waiting list	*-Anxiety: GAD-7* *-Depressive symptoms: PHQ-9* *-Anxiety and depressive symptoms: HADS*	Intervention group had a significantly higher improvement in anxiety (*p* = 0.030) and depressive symptoms (*p* < 0.001). There were also statistically significant time by group interactions for HADS-depressive symptoms (*p* = 0.007), and HADS-anxiety (*p* = 0.001).
**Heapy et al., 2017** **RCT** **USA**	N = 125 57.9 (11.6) yrs 22%F/78%M *Chronic back pain*	CBT Interactive voice response	Face-to-Face CBT	*Depressive symptoms: BDI-II*	There were no significant differences between e-CBT and face-to-face CBT in depressive symptoms.
**Hedman-Lagerlöf et al., 2018** **RCT** **Sweden**	N = 140 50.8 (24--77) yrs 98%F/2%M *Fibromyalgia*	Online exposure therapy Internet-based	Waiting list	*-Depressive symptoms: PHQ-9* *-Anxiety: GAD-7*	There were statistically significant interactions in favor of intervention group for depressive symptoms and anxiety (all, *p* < 0.001).
**Herbert et al., 2017** **RCT** **USA**	N = 128 18%F/82%M 52.0 (13.3) yrs *Unspecific chronic pain*	ACT Video teleconferencing	Face-to-face ACT	*-Depressive symptoms: PHQ-9* *-Pain-related anxiety: PASS-20*	There were no significant differences for any outcomes.
**Hernando-Garijo et al., 2021** **RCT** **Spain**	N = 34 53.4 (8.8) yrs 100%F *Fibromyalgia*	Video-guided aerobic training + usual medical prescription Videoconferencing	Usual medical prescription	*Anxiety and depressive symptoms: HADS*	There was a statistically significant higher improvement in psychological distress (*p* = 0.002) according to HADS than control group.
**Juhlin et al., 2021** **RCT** **Sweden**	N = 139 47.6 (10.1) yrs 90%F/10%M *Chronic widespread pain*	Person-centered intervention supported by online platform Internet-based	Person-centered intervention	*Stress: SCI-93*	No statistically significant differences between groups for stress (*p* = 0.21).
**Lin et al., 2017** **RCT** **Germany**	N = 201 51.0 (12.4) yrs 86%F/14%M *Unspecific chronic pain*	Online guided ACT Internet-based	Waiting list	*Depressive symptoms: PHQ-9* *Anxiety: GAD-7*	There was a significant interaction effect for group x time on depressive symptoms (*p* < 0.05) in favor of intervention group.
**Moessner et al., 2012** **RCT** **Germany**	N = 75 45.9 (9.1) yrs 56%F/44%M *Chronic back pain*	Self-monitoring + Online guided chat Internet-based	Usual care	*Anxiety and depressive symptoms: HADS*	There were no significant differences in other outcomes.
**Peters et al., 2017** **RCT** **Sweden**	N = 284 48.6 (12.0) yrs 85%F/15%M *Chronic back, neck or shoulder pain*	G1: Online Positive psychology G2: Online CBT Internet-based	Waiting list	*Depressive symptoms and Anxiety: HADS*	Both intervention groups showed significant differences with the waiting list group for depressive symptoms (*p* < 0.001). There were also significant differences for anxiety.
**Petrozzi et al., 2019** **RCT** **New Zealand**	N = 108 50.4 (13.6) yrs 50%F/50%M *Chronic LBP*	Online CBT+ Usual care Internet-based	Usual care	*Depressive symptoms, anxiety and stress: DASS-21*	There were no statistically significant differences between the two groups for depressive symptoms (0.98), anxiety (*p* = 0.19) or stress (*p* = 0.41) at any time-points.
**Rickardsson et al., 2021** **RCT** **Sweden**	N = 113 49.5 (12.1) yrs 75%F/25%M *Unspecific chronic pain*	Online ACT Internet-based	Waiting list	*Anxiety: GAD-7* *Depressive symptoms: PHQ-9*	The intervention group showed significant interaction effects of time x group for anxiety (*p* = 0.03) and depressive symptoms (*p* = 0.001).
**Ruehlman et al., 2012** **RCT** **USA**	N = 305 44.9 (N/R) yrs 64%F/36%M *Unspecific chronic pain*	Online self-management Internet-based	Usual care	*-Depressive symptoms: CES-D* *-Depressive symptoms, anxiety and stress: DASS*	Intervention group showed a significant group x time interaction in depressive symptoms (*p* = 0.03 and *p* = 0.04), stress (*p* = 0.00) and anxiety (*p* = 0.05)
**Sander et al., 2020**	N = 295 52.8 (7.7) yrs 62%F/38%M *Unspecific chronic pain*	Online CBT + Usual care Internet-based	Usual Care	*Depressive symptoms: HamD, QIDS score and PHQ-9*	Intervention group had a statistically significant greater improvement of all the outcomes compared with control group.
**Schlickler et al., 2020** **RCT** **Germany**	N = 76 50.8 (7.9) yrs 55%F/45%M *Chronic back pain*	Online CBT-based intervention Internet-based and mobile-based	Waiting list	*-Depressive symptoms: CES-D and QIDS-SR16* *-Anxiety: HamADS*	There was a significant reduction in both treatment in depressive symptoms according to CES-D (*p* < 0.001) with a significant difference in favor of the intervention group post-treatment (*p* = 0.03). Intervention group also showed a significant greater reduction in anxiety (*p* = 0.001).
**Scott et al., 2018** **RCT** **UK**	N = 63 45.5 (14.0) yrs 64%F/36%M *Unspecific chronic pain*	Online ACT + Usual care Internet-based	Usual care	*Depressive symptoms: PHQ-9*	Intervention group showed medium effects on depressive symptoms.
**Shigaki et al., 2013** **RCT** **USA**	N = 108 49.8 (11.9) yrs 94%F/6%M *Rheumatoid arthritis*	Education and social network website about Rheumatoid arthritis + Telephone calls Internet-based	Waiting list	*Depressive symptoms: CES-D*	No statistically significant differences in depressive symptoms (*p* = 0.14).
**Simister al., 2018** **RCT**	N = 67 39.7 (9.4) yrs 95%F/5%M *Fibromyalgia*	Online ACT + Usual care Internet-based	Usual care	*Depressive symptoms: CES-D*	Intervention group significantly improved, relative to control group, on depressive symptoms (*p* = 0.02).
**Smith et al., 2019** **RCT** **Australia**	N = 80 45.0 (13.9) yrs 88%F/12%M *Unspecific chronic pain*	Online self-management and CBT-based intervention Internet-based	Usual care	*Depressive symptoms: PHQ-9*	There was no statistically significant interaction for depressive symptoms.
**Ström et al., 2000** **RCT** **Sweden**	N = 45 36.7 (N/R) yrs 69%F/31%M *Recurrent headache sufferers*	Online relaxation and problem-solving intervention Internet-based	Wait-list	*Depressive symptoms: BDI*	There were no significant differences for depressive symptoms.
**Tavallaei et al., 2018** **RCT** **Iran**	N = 30 33.7 (9.0) yrs 100%F *Migraine and tension-type headache*	Mindfulness-based Stress Reduction Bibliotherapy Internet-based	Usual care	*Depressive symptoms, anxiety and stress: DASS-21*	N/R
**Trompetter et al., 2015** **RCT** **The Netherlands**	N = 238 52.7 (12.4) yrs 76%F/24%M *Unspecific chronic pain*	Online ACT Internet-based	Waiting list	*Depressive symptoms and Anxiety: HADS*	There was a statistically significant difference in depressive symptoms (*p* = 0.006).
**Trudeau et al., 2015** **RCT** **USA**	N = 228 49.9 (11.6) 68%F/32%M *Arthritis*	Online self-management intervention Internet-based	Waiting List	*Depressive symptoms, anxiety, and stress: DASS-21*	No statistically significant condition-by-time effect on the three subscales of the DASS-21.
**Vallejo et al., 2015** **RCT** **Spain**	N = 60 51.6 (9.9) yrs 100%F *Fibromyalgia*	Online CBT + Usual care Internet-based	G1: Face-to-face CBT + Usual care G2: Usual care	*Depressive symptoms and anxiety: HADS* *Depressive symptoms: BDI*	Both groups improved depressive symptoms (both, *p* < 0.01) and HADS scores.
**Westenberg et al., 2018** **RCT** **USA**	N = 126 54.5 (15.0) yrs 50%F/50%M *Upper limb disorders*	Online Mindfulness Internet-based	Attention control	*-Depressive symptoms: N/R* *-Anxiety: N/R*	Intervention group had statistically significant improvements in depressive symptoms (*p* = 0.004) and anxiety (*p* = 0.024).
**Williams et al., 2010** **RCT** **USA**	N = 118 50.5 (11.5) yrs 95%F/5%M *Fibromyalgia*	Online CBT + Usual care Internet-based	Usual care	*-Depressive symptoms: CES-D* *-Anxious mood: STPI—state anxiety*	There were no statistically significant differences in anxiety and depressive symptoms.
**Wilson et al., 2015** **RCT** **USA**	N = 114 49.3 (11.6) yrs 78%F/12%M *Unspecific chronic pain*	Online pain management program Internet-based	Waiting list	*Depressive symptoms: PHQ-9*	There were no statistically significant interactions for group-by-time on depressive symptoms.
**Wilson et al., 2018** **RCT** **USA**	N = 60 44.3 (12.0) yrs 44%F/56%M *Unspecific chronic pain*	Online self-management program Internet-based	Waiting list	*Depressive symptoms: PHQ-8*	Intervention group had higher depressive symptoms score at the end of the intervention (*p* = 0.001).

Abbreviatures: %F: Proportion of women; %M: Proportion of men; ACT: Acceptance and Commitment therapy; BDI: Beck Depression Inventory; BDI-II: Beck Depression Inventory-II, CBT: Cognitive-behavioral therapy; CES-D: Center for Epidemiological Studies Depression Scale; CES-D 10: Center for Epidemiologic Studies Short Depression Scale; DASS: Depression Anxiety Stress Scale; DASS-21: 21-Item Depression Anxiety Stress Scales; GAD-7: 7-Item Generalized Anxiety Disorder; HADS: Hospital Anxiety and Depression Scale; LBP: Low back pain; HamADS: Hamilton Anxiety and Depression Scale; HamD: Hamilton Depression Rating Scale; IRGL: Impact of Rheumatic Diseases on General Health and Lifestyle; N/R: Not reported; PASS-20: 20-item Pain Anxiety Symptoms Scale-Short Form; PHQ-8: 8-Item Personal Health Questionnaire Depression Scale; PHQ-9: 9-Item Personal Health Questionnaire Depression Scale; QIDS: Quick Inventory of Depressive Symptomatology; RCT: Randomized controlled trial; SD: Standard deviation; SCI-93: Stress and Crisis Inventory; STPI: State-Trait Personality Inventory; QIDS-SR16: Quick Inventory of Depressive Symptomatology Self-Report.

### Appendix A.4. Details of the Interventions

**Table A2:** Details of the Interventions.

Authors, Year	Intervention			Comparator		
	Format Equipment and Contact Form	Modality and Content	Duration and Frequency, Follow-Up	Format Equipment	Modality and Content	Duration and Frequency, Follow-Up
**Amorim et al., 2019**	**Mobile application** Written, pedometer Telephone call, message	Physical exercise, activity tracker, lessons - Goal setting (behavior) - Problem solving - Action planning - Social support (emotional) - Instruction on how to perform the behavior - Feedback on outcomes of behavior - Graded tasks	6 months 1 face-to-face interview and 2 calls/month Follow-up: N/A	**Recommendations** Written, brief advice	- Autonomous increase in physical activity - Benefits of physical activity	6 months N/A Follow-up: N/A
**Ang et al., 2010**	**Telephone call + usual care** Written Telephone call	**CBT**. Lessons, relaxation - Action planning - Reduce negative emotions - Framing/reframing	6 weeks 1 session/week Follow-up: 12 weeks	**Usual care**	- Usual treatment by the physician	6 weeks N/A Follow-up: 12 weeks
**Berman et al., 2009**	**Internet-based** Images, audio Email	**Self-care.** Mind–body exercises and lessons - Problem solving - Action planning - Monitoring of behavior by others without feedback - Instruction on how to perform the behavior	6 weeks ≥1 session/week Follow-up: N/A	**No intervention** N/A	N/A	N/A N/A Follow-up: N/A
**Boselie et al., 2018**	**Internet-based** Online platform Telephone call, email	**Positive psychology** exercises - Problem solving - Social support (unspecified) - Instruction on how to perform the behavior	8 weeks Call: weeks 1, 3, 5,7 Email: weeks 2, 4, 6, 8 Follow-up: N/A	**Waiting list** N/A	N/A	N/A N/A Follow-up: N/A
**Bossen et al., 2013**	**Internet-based** Written, video Email	Behavior-graded activity and exercises - Goal setting (behavior) - Instruction on how to perform the behavior - Graded tasks	9 weeks ≥1 session/week Follow-up: 12 weeks	**Waiting list** N/A	N/A	N/A N/A Follow-up: 12 weeks
**Brattberg, 2007**	**Internet-based** Written, video Internet guided chat	Self-help about pain. - Problem solving - Monitoring of emotional consequences - Anticipated regret - Reduce negative emotions	20 weeks 1 video/week Follow-up: 12 months	**Waiting list**	Maintain pharmacotherapy	20 weeks N/A Follow-up: 12 months
**Brattberg, 2008**	**Internet-based** Written Telephone call, email	**Self-management**. Emotional Freedom TechniquesSelf-monitoring of outcome of behavior	8 weeks 1 time/day Follow-up: N/A	**Waiting list**	N/A	N/A N/A Follow-up: N/A
**Bromberg et al., 2012**	**Internet-based** +usual care Written Email	Behavior change, physical activity, lessons - Goal setting (outcome) - Monitoring of behavior by others without feedback - Self-monitoring of behavior - Graded tasks	6 months ≥2 sessions/week (first 4 weeks) ≥1 sessions/month (final 5 month) Follow-up: N/A	**Usual care** N/A	Maintain the routine care and self-management effort	N/A N/A Follow-up: N/A
**Buhrman et al., 2004**	**Internet-based** Slideshow, audio Telephone call	**CBT.** Physical and psychological exercises, relaxation - Goal setting (behavior) - Problem solving - Instruction on how to perform the behavior - Self-monitoring of behavior - Graded tasks	6 weeks 1 call/week Follow-up: 3 months	**Waiting list** N/A	N/A	N/A N/A Follow-up: 3 months
**Buhrman et al., 2011**	**Internet-based** Written Email	**CBT.** Physical exercise, relaxation, cognitive skills - Self-monitoring of behavior	8 weeks N/R Follow-up: 12 weeks	**Waiting list** N/A	N/A	N/A N/A Follow-up: 12 weeks
**Dear et al., 2013**	**Internet-based** Written Telephone call	**CBT.** Lessons, homework - Goal setting (behavior) - Graded tasks	8 weeks 1 lesson/7–10 days 1 call/week Follow-up: 3 months	**Waiting list** N/A	N/A	N/A N/A Follow-up: 3 months
**Dear et al., 2015**	**Internet-based** - G1: CBT + Regular online contact - G2: CBT + optimal online contact - G3: CBT Slideshow Telephone call, email	**CBT.** Lessons, homework - Problem solving - Instruction on how to perform the behavior - Behavioral practice - Graded tasks	8 weeks 1 lesson/7–10 days G1: 1 call/week G2: as-needed calls G3: no contact Follow-up: 3 months	**Waiting list** N/A	N/A	N/A N/A Follow-up: 3 months
**Devineni and Blanchard, 2005**	**Internet-based** Written, audio, web pages Email	Lessons, exercises, relaxation, Behavioral headache-related intervention Autogenic training - Self-monitoring of outcome - Reduce negative emotions	4 weeks N/R Follow-up: 2 months	**Waiting list**	N/A	N/A N/A Follow-up: 2 months
**Ferwerda et al., 2017**	**Internet-based** Written Email	**CBT.** Lessons, homework - Goal setting (behavior) - Problem solving - Action planning - Instruction on how to perform the behavior - Reduce negative emotions - Distraction - Framing/reframing	17 to 32 weeks 1 email/1–2 weeks Follow-up: 12 months	**Usual care** N/R	Rheumatological care	N/R N/R Follow-up: 12 months
**Friesen et al., 2017**	**Internet-based** Slideshow Telephone call, email	**CBT.** Lessons, homework - Problem solving - Feedback on perform the behavior - Instruction on how to perform the behavior	8 weeks 1 email and call/week Follow-up: N/A	**Waiting list** N/A	N/A	N/A N/A Follow up: N/A
**Heapy et al., 2017**	**Interactive voice response** Written, images, audio, pedometer Telephone call	**CTB.** Lessons, relaxation - Goal setting (outcome) - Feedback on behavior - Graded tasks - Reduce negative emotions	10 weeks 1 call/day Follow-up: 9 months	**Face-to-face** Written, images, audio, pedometer	**CBT.** Lessons, relaxation - Goal setting (outcome) - Feedback on behavior - Graded tasks - Reduce negative emotions	10 weeks 1 session/week Follow-up: 9 months
**Hedman-Lagerlöf et al., 2018**	**Internet-based** Written Telephone call, message	Lessons, homework, mindfulness - Goal setting (behavior) - Problem solving - Monitoring of behavior by others without feedback - Exposure - Graded tasks	10 weeks 1–3 contact/week Follow-up: 12 months	**Waiting list** N/A	N/A	N/A N/A Follow-up: 12 months
**Herbert et al., 2017**	V**ideoconferencing** Written N/R	**ACT.** Mindfulness, lessons - Goal setting - Information about emotional consequences	8 weeks 1 session/week Follow-up: 6 months	**Face-to-face** Written	ACT. Mindfulness, lessons - Goal setting - Information about emotional consequences	8 weeks 1 session/week Follow-up: 6 months
**Hernando-Garijo et al., 2021**	**Videoconferencing + usual** care Video Video call	Aerobic exercise - Low-impact exercise	15 weeks 2 session/week Follow-up: N/A	**Usual care** N/A	- Maintain pharmacotherapy	15 weeks N/A Follow-up: NA
**Juhlin et al., 2021**	**Internet-based** Digital platform Message	**Person-centered intervention.** Physical and psychological exercises - Goal setting (behavior) - Problem solving - Action planning	6 months 1 contact/week Follow-up: N/A	**Face-to-face** **(1 session)** N/A	- **Person-centered intervention.** Physical and psychological exercises	6 months N/A Follow-up: N/A
**Lin et al., 2017**	**Internet-based** Written, audio, video Email, message	**ACT.** Lessons, mindfulness - Goal setting (behavior) - Reduce negative emotions	9 weeks 1 session/week Follow-up: 6 months	**Waiting list** N/A	- N/A	N/A N/A Follow-up: 6 months
**Moessner et al., 2012**	**Internet-based** N/R Internet guided chat	**Self-monitoring.** Lessons - Self-monitoring of behavior - Behavioral practice/rehearsal	12–15 weeks 1 session/week Follow-up: 6 months	**Usual care** N/A	N/R	12–15 weeks 1 session/week Follow-up: 6 months
**Peters et al., 2017**	**Internet-based** Written Telephone call, email	G1: **Positive psychology.** Psychological exercises - Goal setting (behavior) - Graded tasks - Reduce negative emotions G2: **CBT.** Lessons, homework, relaxation - Problem solving - Action planning - Social support (unspecified) - Framing/reframing	8 weeks 1 lesson/week Call: weeks 1, 3, 5, 7 Email: weeks: 2, 4, 6, 8 Follow-up: 6 months	**Waiting list** N/A	N/A	N/A N/A Follow-up: 6 months
**Petrozzi et al., 2019**	**Internet-based + usual care** Written Telephone call	**CBT.** Lessons, homework - Problem solving - Self-monitoring behavior - Instruction on how to perform the behavior - Distraction	8 weeks 1 lesson/week 1 call/week Follow-up: 12 months	**Usual care** N/A	- Physical treatment (manual therapy, exercise and/or education) - Recommendation for physical activity	8 weeks 12 sessions (variable frequency) Follow-up: 12 months
**Rickardsson et al., 2021**	**Internet-based** Written, image, audio Telephone call, message	**ACT.** Lessons - Instruction on how to perform the behavior - Feedback on behavior - Graded tasks - Non-specific reward - Distraction	8 weeks 7 sessions/week ≥2 messages/week Follow-up: 12 months	**Waiting list** N/A	- Maintain usual treatment	N/A N/A Follow-up: 12 months
**Ruehlman et al., 2012**	**Internet-based** Written, image Email, message	**Self-management + e-community.** Physical exercise, lessons, homework, relaxation - Goal setting (outcome) - Action planning - Self-monitoring of outcome of behavior - Instruction on how to perform the behavior - Reduce negative emotions	6 weeks N/R Follow-up: 14 weeks	**Usual care** N/A	N/R	6 weeks N/A Follow-up: 14 weeks
**Sander et al., 2020**	**Internet-based + usual care** Written, audio, video Telephone call, email, message	**CBT.** Lessons, homework, relaxation - Problem solving - Action planning - Feedback on behavior - Reduce negative emotions	9 weeks 7 sessions/week Follow-up: 12 months	**Usual care** N/A	Medical or psychological treatment	9 weeks N/R Follow-up: 12 months
**Schlickler et al., 2020**	**Internet-based + mobile-based** N/R Email, message	**CBT.** Lessons, mindfulness, relaxation - Problem solving - Feedback on behavior - Social support - Non-specific reward - Reduce negative emotions - Framing/reframing	9 weeks 7 lessons/week Follow-up: 6 months	**Waiting list** N/A	N/A	N/A N/A Follow-up: 6 months
**Scott et al., 2018**	**Internet-based + usual care** Video Telephone call, email	**ACT.** Lessons - Goal setting (behavior) - Feedback on behavior - Instruction on how to perform the behavior - Monitoring of emotional consequences	5 weeks 2 lesson/week (first 3 weeks), 1 lesson/week (final 2 weeks) Follow-up: 9 months	**Usual care** N/A	- Medical treatment - Instruction on how to perform the behavior	5 weeks N/A Follow-up: 9 months
**Shigaki et al., 2013**	**Internet-based** Slideshow Telephone call, message, online chat	Lessons, homework - Problem solving - Self-monitoring behavior	10 weeks 1 lesson/week 1 call/week Follow-up: N/A	**Waiting list**	- N/A	N/A N/A Follow-up: N/A
**Simister al., 2018**	**Internet-based + usual care** Written, audio, video Email	**ACT.** Lessons, homeworkFeedback on behaviorNon-specific reward	8 weeks N/R Follow-up: 3 months	**Usual care** N/A	- Maintain usual treatment	8 weeks N/A Follow-up: 3 months
**Smith et al., 2019**	**Internet-based** Written, image, audio, video Telephone call, email	**CBT and self-management.** Multidisciplinary program with physical exercise, lessons, homework, relaxation - Goal setting (behavior and outcome) - Problem solving - Instruction on how to perform the behavior - Graded tasks - Multidisciplinary program - Physical therapy, psychologist	4 months 2 lessons/month Follow-up: 7 months	**Usual care** N/A	- Maintain usual treatment	4 months N/A Follow-up: 7 months
**Ström et al., 2000**	**Internet-based** Written Email	Lessons, relaxation - Problem solving - Instruction on how to perform the behavior - Feedback on outcome of behavior	6 weeks 1 lesson/week Follow-up: N/A	**Waiting list** N/A	N/A	N/A N/A Follow-up: N/A
**Tavallaei et al., 2018**	**Internet-based** Written N/R	**Mindfulness**-based stress reduction bibliotherapy - Problem solving - Action planning - Distraction	8 weeks 1 lesson/week Follow-up: N/A	**Usual care** N/A	- Pharmacotherapy	8 weeks N/A Follow-up: N/A
**Trompetter et al., 2015**	**Internet-based** Written Email	**ACT.** Lessons, mindfulness - Self-monitoring of behavior - Non-specific reward - Distraction	3 months ≥3 h/week Follow-up: 6 months	**Waiting list** N/A	N/A	N/A N/A Follow-up: 6 months
**Trudeau et al., 2015**	**Internet-based** Multimedia materials Telephone call, email	**Self-management.** Lessons - Problem solving - Instruction on how to perform the behavior - Reduce negative emotions	6 months ≥2 sessions/week (1 month) 1 session/month (5 months) Follow-up: N/A	**Waiting list** N/A	N/A	N/A N/A Follow-up: N/A
**Vallejo et al., 2015**	**Internet-based + usual care** Written, images, audio Message	**CBT.** Lessons, homework, relaxation - Problem solving - Feedback on behavior - Reduce negative emotions - Framing/reframing	10 weeks 1 session/week Follow-up: 12 months	G1: **Face-to-face + usual care** Written, images, audio G2: **Usual care** N/A	G1: **CBT.** Lessons, homework, relaxation - Problem solving - Reduce negative emotions - Framing/reframing G2: Pharmacotherapy	10 weeks G1: 1 session/week G2: N/A Follow-up (only G1): 12 months
**Westenberg et al., 2018**	**Internet-based** Written, video N/R	Mindfulness - Reduce negative emotions	60-s video N/R Follow-up: N/A	**Attention control** Written	- Health information	60-s read N/R Follow-up: N/A
**Williams et al., 2010**	**Internet-based + usual care** Written, audio, video No contact	**Self-management.** Lessons, homework, relaxation - Goal setting (behavior) - Problem solving - Self-monitoring of behavior - Social support (unspecified) - Instruction on how to perform the behavior - Graded tasks - Framing/reframing	6 months N/R Follow-up: N/A	**Usual care**	- Maintain usual treatment from care physician	6 months N/A Follow-up: N/A
**Wilson et al., 2015**	**Internet-based** N/R N/R	**Self-management.** Lessons, exercises, relaxation - Goal setting (outcome) - Self-monitoring or outcome of behavior	8 weeks N/R Follow-up: N/A	**Usual care** N/A	N/A	8 weeks N/R Follow-up: N/A
**Wilson et al., 2018**	**Internet-based** Written Interactive activity	**Self-management.** Lessons, homework - Self-monitoring of behavior - Behavioral practice/rehearsal	8 weeks N/R Follow-up: N/A	**Waiting list** Written	- Educational tips	8 weeks 1 email/week Follow-up: N/A

ACT: Acceptance and Commitment therapy; CBT: Cognitive-behavioral therapy; N/A: Not applicable; N/R: Not reported; NSAIDs: Nonsteroidal anti-inflammatory drugs.

### Appendix A.5. Assessment of the Quality of the Studies Based on the PEDro Scale

**Table A3:** PEDro scale.

Items												
Articles	1	2	3	4	5	6	7	8	9	10	11	Total
**Amorim et al., 2019**	1	1	1	1	0	0	1	0	1	1	1	**7**
**Ang et al., 2010**	1	1	0	1	0	0	1	1	0	1	1	**6**
**Berman et al., 2009**	1	1	0	1	0	0	0	1	0	1	1	**5**
**Boselie et al., 2018**	0	1	0	1	0	0	0	0	0	1	1	**4**
**Bossen et al., 2013**	1	1	1	1	0	0	0	0	1	1	1	**6**
**Brattberg, 2007**	1	1	1	1	0	0	0	1	1	1	1	**7**
**Brattberg, 2008**	1	1	1	1	0	0	0	1	1	1	1	**7**
**Bromberg et al., 2012**	1	1	0	1	0	0	0	1	1	1	1	**6**
**Buhrman et al., 2004**	1	1	0	1	0	0	0	1	0	1	1	**5**
**Buhrman et al., 2011**	1	1	1	1	0	0	0	1	1	1	1	**7**
**Dear et al., 2013**	1	1	0	1	0	0	0	1	0	1	1	**5**
**Dear et al., 2015**	1	1	1	1	0	0	0	1	0	1	1	**6**
**Devineni and Blanchard, 2005**	1	1	1	1	0	0	0	1	0	1	1	**6**
**Ferwerda et al., 2017**	1	1	1	1	0	0	0	1	1	1	1	**7**
**Friesen et al., 2017**	1	1	1	1	0	0	0	1	0	1	1	**6**
**Heapy et al., 2017**	1	1	1	1	0	0	0	0	1	1	1	**6**
**Hedman-Lagerlöf et al., 2018**	1	1	1	1	0	0	0	1	0	1	1	**6**
**Herbert et al., 2017**	1	1	0	1	0	0	1	1	1	1	1	**7**
**Hernando-Garijo et al., 2021**	1	1	0	1	0	0	1	1	1	1	1	**7**
**Juhlin et al., 2021**	1	1	1	1	0	0	0	0	1	1	1	**6**
**Lin et al., 2017**	1	1	1	1	0	0	0	0	1	1	1	**6**
**Moessner et al., 2012**	1	1	0	1	0	0	0	0	1	1	1	**5**
**Peters et al., 2017**	1	1	0	1	0	0	0	0	1	1	1	**5**
**Petrozzi et al., 2019**	1	1	1	1	0	0	0	1	1	1	1	**7**
**Rickardsson et al., 2020**	1	1	1	1	0	0	0	1	1	1	1	**7**
**Ruehlman et al., 2012**	1	1	0	1	0	0	0	0	1	1	1	**5**
**Sander et al., 2020**	1	1	1	1	0	0	1	0	1	1	1	**7**
**Schlicker et al., 2021**	1	1	0	1	0	0	0	1	1	1	1	**6**
**Scott et al., 2018**	1	1	1	1	0	0	0	1	1	1	1	**7**
**Shigaki et al., 2013**	1	1	0	0	0	0	0	1	0	1	1	**4**
**Simister et al., 2018**	1	1	1	1	0	0	0	1	1	1	1	**7**
**Smith et al., 2019**	1	1	0	1	0	0	1	0	1	1	1	**6**
**Ström et al., 2000**	1	1	0	1	0	0	0	0	1	1	1	**5**
**Tavallaei et al., 2018**	1	1	0	0	0	0	0	1	0	1	1	**4**
**Trompetter et al., 2014**	1	1	0	1	0	0	0	1	1	1	1	**6**
**Trudeau et al., 2015**	1	1	1	1	0	0	0	1	1	1	1	**7**
**Vallejo et al., 2015**	1	1	0	1	0	0	0	1	1	1	1	**6**
**Westenberg et al., 2018**	1	1	0	1	1	0	0	1	1	1	1	**7**
**Williams et al., 2010**	1	1	1	1	0	0	0	1	1	1	1	**7**
**Wilson et al., 2015**	1	1	0	1	0	0	0	0	1	1	1	**5**
**Wilson et al., 2018**	1	1	0	1	1	0	0	1	1	1	1	**7**

Notes: 1: subject choice criteria are specified; 2: random assignment of subjects to groups; 3: hidden assignment; 4: groups were similar at baseline; 5: all subjects were blinded; 6: all therapists were blinded; 7: all evaluators were blinded; 8: measures of at least one of the key outcomes were obtained from more than 85% of baseline subjects; 9: intention-to-treat analysis was performed; 10: results from statistical comparisons between groups were reported for at least one key outcome; 11: the study provides point and variability measures for at least one key outcome.
